# Self-assembling asymmetric peptide-dendrimer micelles – a platform for effective and versatile *in vitro* nucleic acid delivery

**DOI:** 10.1038/s41598-018-22902-9

**Published:** 2018-03-19

**Authors:** Ganesh R. Kokil, Rakesh N. Veedu, Bao Tri Le, Grant A. Ramm, Harendra S. Parekh

**Affiliations:** 1 School of Pharmacy, Pharmacy Australia Centre of Excellence, The University of Queensland, Brisbane, QLD Australia; 2 Center for Comparative Genomics, Murdoch University, Murdoch, WA Australia; 3 Western Australian Neuroscience Research Institute, Perth, WA Australia; 4 School of Chemistry and Molecular Biosciences, The University of Queensland, Brisbane, QLD Australia; 5 The Hepatic Fibrosis Group, QIMR Berghofer Medical Research Institute, Brisbane, QLD Australia; 6 Faculty of Medicine and Biomedical Sciences, The University of Queensland, Brisbane, QLD Australia

## Abstract

Despite advancements in the development of high generation cationic-dendrimer systems for delivery of nucleic acid-based therapeutics, commercially available chemical agents suffer from major drawbacks such as cytotoxicity while being laborious and costly to synthesize. To overcome the aforementioned limitations, low-generation cationic peptide asymmetric dendrimers with side arm lipid (cholic and decanoic acid) conjugation were designed, synthesized and systematically screened for their ability to self-assemble into micelles using dynamic light scattering. Cytotoxicity profiling revealed that our entire asymmetric peptide dendrimer library when trialled alone, or as asymmetric dendrimer micelle-nucleic acid complexes, were non-cytotoxic across a broad concentration range. Further, the delivery efficiency of asymmetric peptide dendrimers in H-4-II-E (rat hepatoma), H2K (mdx mouse myoblast), and DAOY (human medulloblastoma) cells demonstrated that cholic acid-conjugated asymmetric dendrimers possess far superior delivery efficiency when compared to the commercial standards, Lipofectamine 2000 or Lipofectin^®^.

## Introduction

Dendrimers by virtue of their structure possess flexible surface functionality, which influences their capacity to complex and/or conjugate therapeutic agents, making them attractive as carriers for drugs/genes^1^. From around the mid-1980’s researchers have primarily focused on improving synthetic approaches to dendrimers, with the aim of enhancing their physicochemical properties and biocompatibility^2^. In this context, high generation spherical dendrimers have been the center of attention, although they are invariably cytotoxic due to their high charge, and are also rapidly cleared, which limits their applicability *in vivo*^3,4^. Another, crucial limitation, often sidelined is the inability to site-specifically functionalize spherical dendrimers with targeting moieties, as the array of surface groups, often amines, have comparable reactivity^5^. Amino acid-based dendrimers also referred to as peptide dendrimers are radial or wedge-like branched macromolecules consisting of a peptidyl core and/or covalently attached surface functional units. Thus, the focus of this research was to address each of these shortfalls, which has led to the development of biocompatible, low generation, asymmetric peptide dendrimers prepared by solid phase peptide synthesis (SPPS)^6^, which now serve as promising substitutes to commercially available spherical dendrimers.

Dendrimer systems synthesized for the ultimate goal of gene delivery and transfection comprise a range of characteristics essential for their purpose, these include:

1. An array of cationic head groups for effective electrostatic interaction with, and condensation of, anionic genes, *e*.*g*., DNA or RNA^7^;
2. A hydrophobic core to promote cell membrane association/partitioning^8^;
3. Targeting ligands for cell-specific recognition, binding and internalization of cargo^9^.

**Figure 1:**
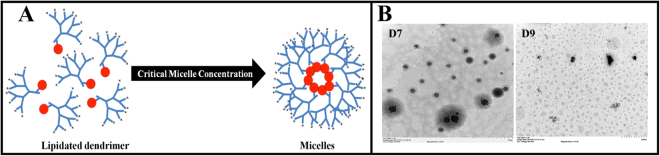
(**A**) Schematic representation of a lipidated (red ball) asymmetric peptide dendrimer (blue branches) micelle forming above its critical micelle concentration (CMC) (**B**) Representative TEM images of D7 and D9, showing morphology characterization of the self-assembled micelles.

To elaborate on each of the features listed above: the cationic charge on dendrimers serves to condense nucleic acids into toroidal structures (in the case of pDNA)^10,11^ or less well-defined structures where relatively low MW genetic material, such as siRNA, is employed^12^. A hydrophobic core will facilitate dendrimer association to the cell membrane^8^. Furthermore, various ligands, e.g. folic acid^13^, RGD-peptide^14^, carbohydrates^15^ and lipids^16^, have been proposed to enhance gene delivery to target cells. Of these targeting ligands, lipid conjugation among the most popular strategies utilized to enhance the physico-chemical characterization of dendrimers. Several attempts have been made to covalently combine dendrimers with lipids to establish a hybrid system for enhanced nucleic acid delivery with improved safety^17,18^. Such dendrimer-lipid hybrid systems are well known to enhance nucleic acid transfection efficiency *in vitro*, as their amphiphilic properties permit electrostatic interaction with anionic nucleic acids while also promoting cell membrane association and internalization^7,8^. In addition, lipids can serve as targeting ligands enhancing cell-specific recognition, binding and cargo internalization^19,20^. Furthermore, such hybrid systems are purported to promote self-assembly into secondary structures, such as micelles (Fig. 1), which provide added protection to therapeutics, such as nucleic acids from the abundance of exo- and endonucleases^21^.

Keeping the aforementioned features firmly in mind, our goal was to synthesize appropriately functionalized low-generation asymmetric peptide dendrimers with various lipid scaffolds that would serve to enhance targeting, cell membrane delivery and partitioning, and lastly facilitate transfection of the nucleic acid cargo. Of the various targeting ligands reported in the literature and elaborated below, two were selected for tethering to our asymmetric dendrimer system, *i*.*e*., cholic acid (CA) and decanoic acid (DA). Our key criteria for selecting these two targeting ligands were (1) biocompatibility, (2) affinity for the target cells/tissue, and (3) appropriate functionality for ease of conjugation to the asymmetric dendrimers i.e. acid or amine-terminated.

**Figure 2:**
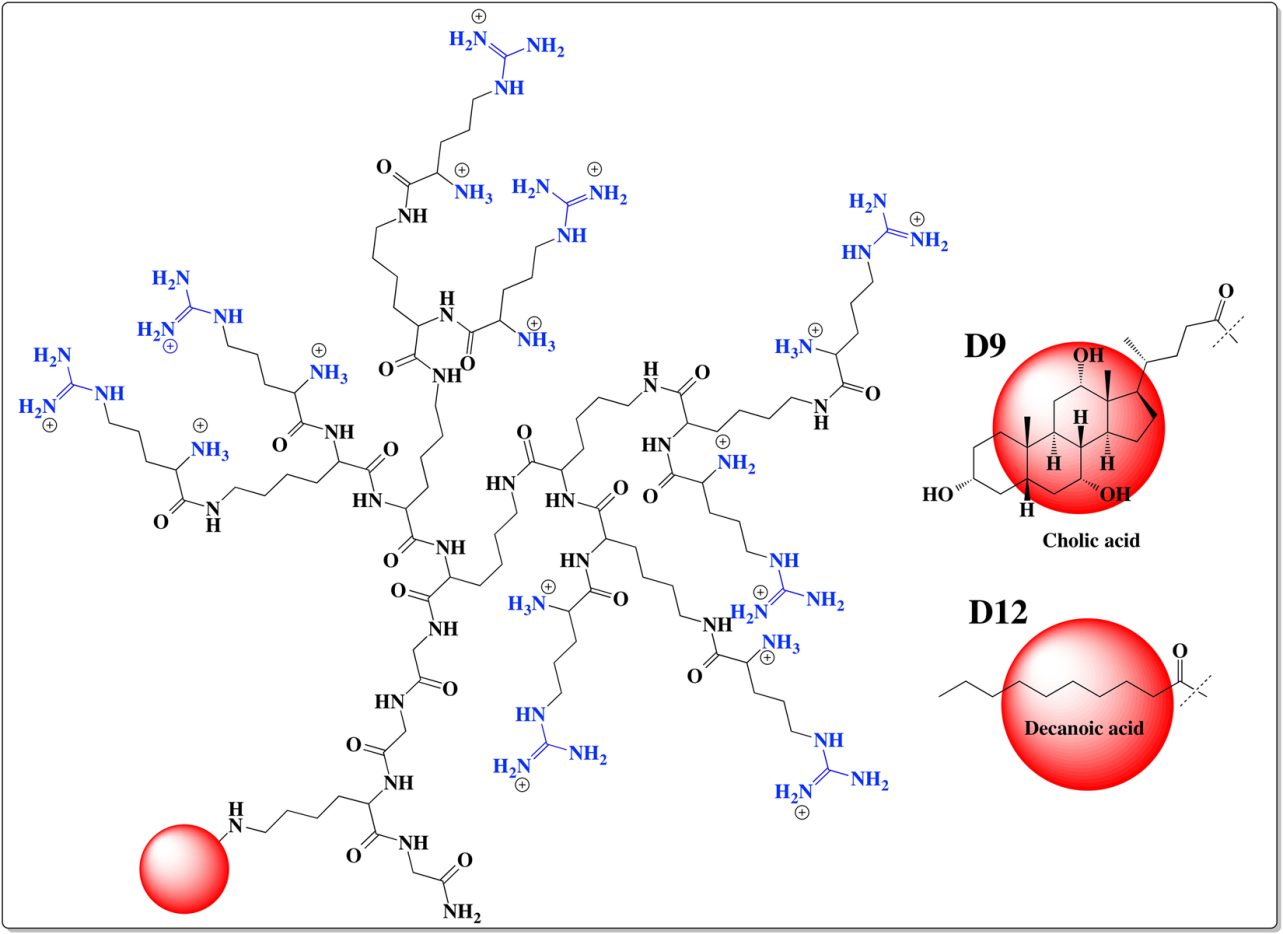
Chemical structures of asymmetric peptide dendrimers. 16^+^ Arg-CA (D9), 16^+^ Arg-DA (D12).

CA, a naturally occurring bile acid with amphiphilic properties has been proven to enhance gastrointestinal absorption while also possessing liver targeting properties^22,23^. Thus, in the present study we constructed CA-containing dendrimers, which were shown to self-assemble into micelles at low micromolar concentrations^22^. Similarly, DA-conjugated^24^ peptides have been reported to possess greater affinity for various cancer cells, which led us to trial this as an alternative dual targeting and self-assembling lipid, alongside CA. Using SPPS^6^, a panel of asymmetric peptide dendrimers, varying in generation and head group chemistry, in the presence or absence of lipid (CA and DA) conjugation were synthesized and characterized as illustrated in Fig. 2 (see S1 Fig for complete library**)**. The library of asymmetric dendrimers varied in charge density (as 4+, 8+ and 16+) and head group (lysine or arginine) chemistry, being synthesized using well-established solid phase peptide synthesis (SPPS) strategies. To enhance nucleic acid delivery *in vitro* a subset of asymmetric dendrimers were prepared with side arm cholic acid or decanoic acid conjugation.

## Results and Discussion

### Asymmetric peptide dendrimers synthesis and purification

The library of low-generation asymmetric peptide dendrimers (Fig. 1, S1 Fig) with (D7 to D12, Arg head group) or without (D1 to D6, Arg or Lys head group) side chain lipid (CA or DA) functionalization, and with 4^+^, 8^+^ or 16^+^ terminal charge, were synthesized using the well-established Fmoc-SPPS protocol^6^. After purification (using preparative RP-HPLC), all asymmetric dendrimers were obtained in moderate-good yield (45 to 66%), calculated from 100 mg of crude sample. Single peak purity of synthesized asymmetric dendrimers were confirmed using analytical RP-HPLC (Table 1), with the accurate mass of each dendrimer confirmed by high resolution-mass spectrometry (HR-MS). The observed molecular ion and R_*t*_ of all dendrimers were shown in Table 1.

**Table 1:** HR-MS (*m/z*) and RP-HPLC (R_t_) data for asymmetric peptide dendrimer library.

Asymmetric peptide dendrimer (Head group charge)	Yield (% recovery)^*^	Molecular formula	Monoisotopic mass	Observed *m/z*	R_t_ (min)
D1 (4^+^)	55.54	C_20_H_42_N_8_O_4_	458.3329	459.3383 [M + H]^+^ 230.1730 [M + 2 H]^2+^	8.20
D2 (8^+^)	60.11	C_44_H_90_N_16_O_8_	970.7128	971.7164 [M + H]^+^ 486.3618 [M + 2 H]^2+^	8.59
D3 (16^+^)	63.07	C_92_H_186_N_32_O_16_	1995.4725	1996.4895 [M + H]^+^ 998.7428 [M + 2H]^2+^ 250.4408 [M + 8 H]^8+^	9.43
D4 (4^+^)	54.35	C_20_H_42_N_12_O_4_	514.3452	515.3506 [M+H]^+^ 258.1769 [M + 2 H]^2+^	8.04
D5 (8^+^)	52.98	C_44_H_90_N_24_O_8_	1082.7373	1083.7543 [M+H]^+^ 361.9197 [M + 3 H]^3+^ 271.6886 [M + 4 H]^4+^	9.17
D6 (16^+^)	45.78	C_92_H_186_N_48_O_16_	2219.5216	1101.7757 [M+2H]^2+^ 444.9148 [M + 5 H]^5+^ 318.0837[M + 7 H]^7+^	10.04
D7 (4^+^)	52.57	C_54_H_98_N_16_O_11_	1146.7601	1447.7762[M+H]^+^, 287. 6952 [M+4H]^4+^	14.57
D8 (8^+^)	55.33	C_78_H_146_N_28_O_15_	1715.1522	858.5883 [M+2 H]^2+^ 572.7229 [M + 3 H]^3+^ 344.0398 [M+5 H]^5+^	13.67
D9 (16^+^)	58.24	C_126_H_242_N_52_O_23_	2852.9399	1427.4788 [M+2 H]^2+^ 571.5973 [M + 5 H]^5+^ 357.6267 [M + 8 H]^8+^	9.40
D10 (4^+^)	54.15	C_40_H_78_N_16_O_8_	910.6189	911.6237 [M+H]^+^ 456.3173 [M + 2 H]^2+^	13.62
D11 (8^+^)	57.23	C_64_H_126_N_28_O_12_	1479.0110	1480.0252 [M + H]^+^ 740.5130[M+2 H]^2+^ 296.8184[M + 5 H]^5+^	13.05
D12 (16^+^)	50.89	C_112_H_222_N_52_O_20_	2616.7987	1309.4097 [M+2H]^2+^ 655.2099 [M + 4 H]^4+^	9.40

^*^Percentage recovery calculated from 100 mg of crude sample.

The overarching aim of the current research was to design, synthesize and fully characterize a panel of low generation asymmetric peptide dendrimers, then assess their performance in delivering gene-based cargo to a range of cells types. These low generation asymmetric peptide dendrimers are not only easily engineered to be devoid of cytotoxicity but also provide site-specific tethering of targeting ligands on their side arm. They possess numerous advantages over commercially available dendrimers, providing a wide range of tunable properties including chirality, hydrophilicity/hydrophobicity and biocompatibility. Our earlier works have shown such dendrimer systems to be non-cytotoxic, and capable of effectively conjugating antibodies, and in that context were found to be efficient in site-specific delivery of DNA to B cells *in vitro* and *in vivo*^10,11^. Furthermore, express *in vitro* plasmid transfection was also achieved with these asymmetric peptide dendrimer systems^25^. Here, to demonstrate the ability of such versatile asymmetric dendrimer systems to deliver nucleic acids *in vitro*, we synthesized a panel of traditional and lipidated asymmetric peptide dendrimers. Functionalization of these asymmetric dendrimers were achieved using various lipid scaffolds (CA and DA) each possessing a carboxylic functionality, which allowed ready tethering to dendrimers via SPPS^6^.

### Lipidated asymmetric peptide dendrimers self-assemble into micelles

**Figure 3:**
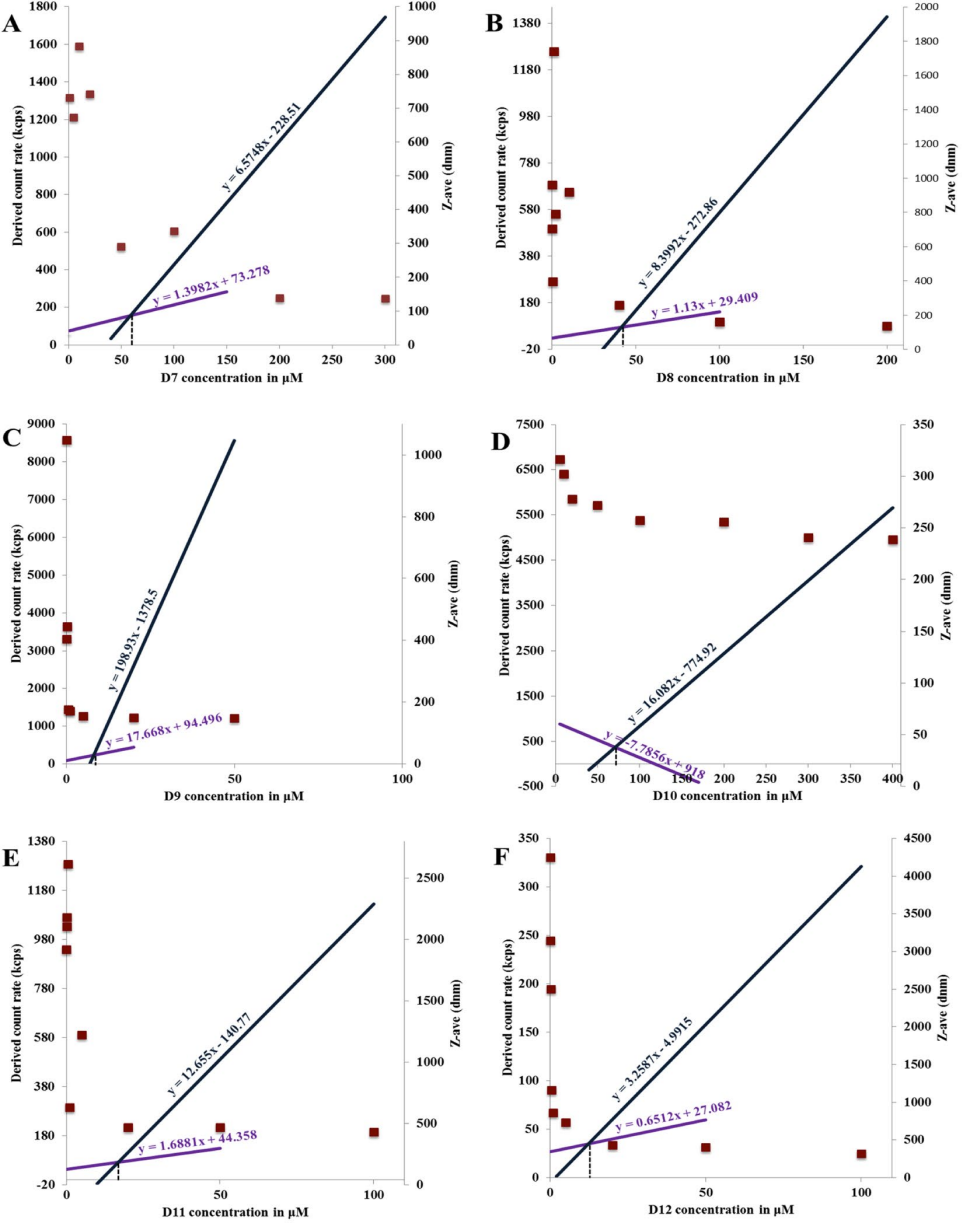
A comparative plot of intensity of scattered light (left axis) and *Z*-ave (right axis) as a function lipidated asymmetric peptide dendrimer (D7-D12) concentrations.

The self-assembling capabilities of side-chain lipidated asymmetric peptide dendrimers D7 to D12, commonly referred to as the critical micelle concentration (CMC), were tracked by dynamic light scattering (DLS). With aqueous solutions of D7 to D12 prepared within a concentration range of 50 nM to 200 μM, intensity values of scattered light in kilo counts per second (kcps) were recorded as a function of dendrimer concentration (Fig. 3A–F).

The scattering intensities detected for lipidated asymmetric peptide dendrimers concentrations below the CMC have a near constant value corresponding to that of deionized water. The intensity started to show a linear increase with concentration at the CMC, since the number of micelles increased in the solution. The intersection of best-fit lines drawn through the data points corresponds to the CMC value of lipidated asymmetric peptide dendrimers^26^. The Z-average (Z-ave) is the intensity weighted mean hydrodynamic size of the ensemble of particles measured by dynamic light scattering (DLS). The hydrodynamic diameter (Z-ave) of resulting lipidated asymmetric peptide dendrimer micelles was also determined by DLS. The *Z*-ave remained constant at concentrations close to the CMC, however, it was not possible to obtain *Z*-ave values when the solution was diluted below the CMC, indicating that self-assembly into secondary structures was not promoted below this critical concentration. Fig. 3 shows the size change as a function of the lipidated asymmetric peptide dendrimer concentration (along the right axis). The plot is arranged such that the intensity and size change dependent on concentration are shown together, with the hydrodynamic diameter of micelles displaying a distinct transition as the CMC is reached.

Table 2 lists the observed CMC values achieved with D7 to D12, with the *Z*-ave determined from the highest concentration used in the study. All lipid-conjugated asymmetric peptide dendrimers (D7 to D12) demonstrated self-assembling properties, forming micelles at concentrations ranging from 8.01 μM, for D9, to 70.92 μM for D10. The *Z*-ave of lipidated asymmetric peptide dendrimers was in the range of 137.2 ± 1.21 nm, for D7 to 457.53 ± 9.29 nm, for D11, with CA conjugated 16^+^ asymmetric peptide dendrimer (D9) showing the lowest CMC value at 8.01 μM and a *Z-*ave of 155.07 ± 4.80 nm **(**Fig. 3C).

**Table 2:** CMC, *Z*-ave and zeta potential of asymmetric peptide dendrimers.

Asymmetric peptide dendrimer	CMC (μM)	Avg. *Z*-ave (d.nm)	Poly dispersity index (PDI)	Avg. Zeta potential
D1	MND	N/A	N/A	16.03 ± 0.15
D2	MND	N/A	N/A	18.10 ± 0.26
D3	MND	N/A	N/A	35.96 ± 0.70
D4	MND	N/A	N/A	9.94 ± 0.77
D5	MND	N/A	N/A	13.77 ± 0.74
D6	MND	N/A	N/A	24.68 ± 0.07
D7	58.30	137.2 ± 1.21	0.274	—
D8	41.58	137.60 ± 2.31	0.249	—
D9	8.01	155.07 ± 4.80	0.262	—
D10	70.92	238.73 ± 2.87	0.300	—
D11	16.88	457.53 ± 9.29	0.431	—
D12	12.30	316.37 ± 3.82	0.356	—

MND – micelles not detected; N/A – not applicable.

### Assessment of zeta potential

The average zeta potential (mV) values ranged from + 24 to + 36 mV for 16^+^ asymmetric peptide dendrimers D3 and D6. Interestingly, there was considerable overlap in the zeta potential values for the 8^+^ (D2, D5; 13 to 18 mV) and 4^+^ (D1, D4; 9–16 mV) systems, when measured at pH 6.6–6.8 (Table 2). A notable increase in the measured zeta potential values of 4^+^/8^+^ and 16^+^ asymmetric peptide dendrimers were observed with the quadrupling/doubling of cationic charged (NH_3_^+^) surface head groups.

### Asymmetric peptide dendrimers form stable complexes with siRNA

The ability of asymmetric peptide dendrimers to form stable complexes with siRNA was confirmed using a gel retardation assay^27^. The assay is based on simple mobility and fluorescence-driven ethidium bromide intercalation of siRNAs under the influence of an electric current. Increasing the concentration of asymmetric peptide dendrimers relative to siRNA (*e*.*g*., 1:1, 2:1, 5:1, 10:1, 20:1, 50:1, 100:1 and 200:1) resulted in gradually weaker visual bands eventually leading to complete loss of fluorescence, which was indicative of full complexation. Loss of fluorescence can be attributed to ejection of ethidium bromide from siRNA as the latter condenses and complexes with increasing concentrations of asymmetric peptide dendrimers.

**Figure 4:**
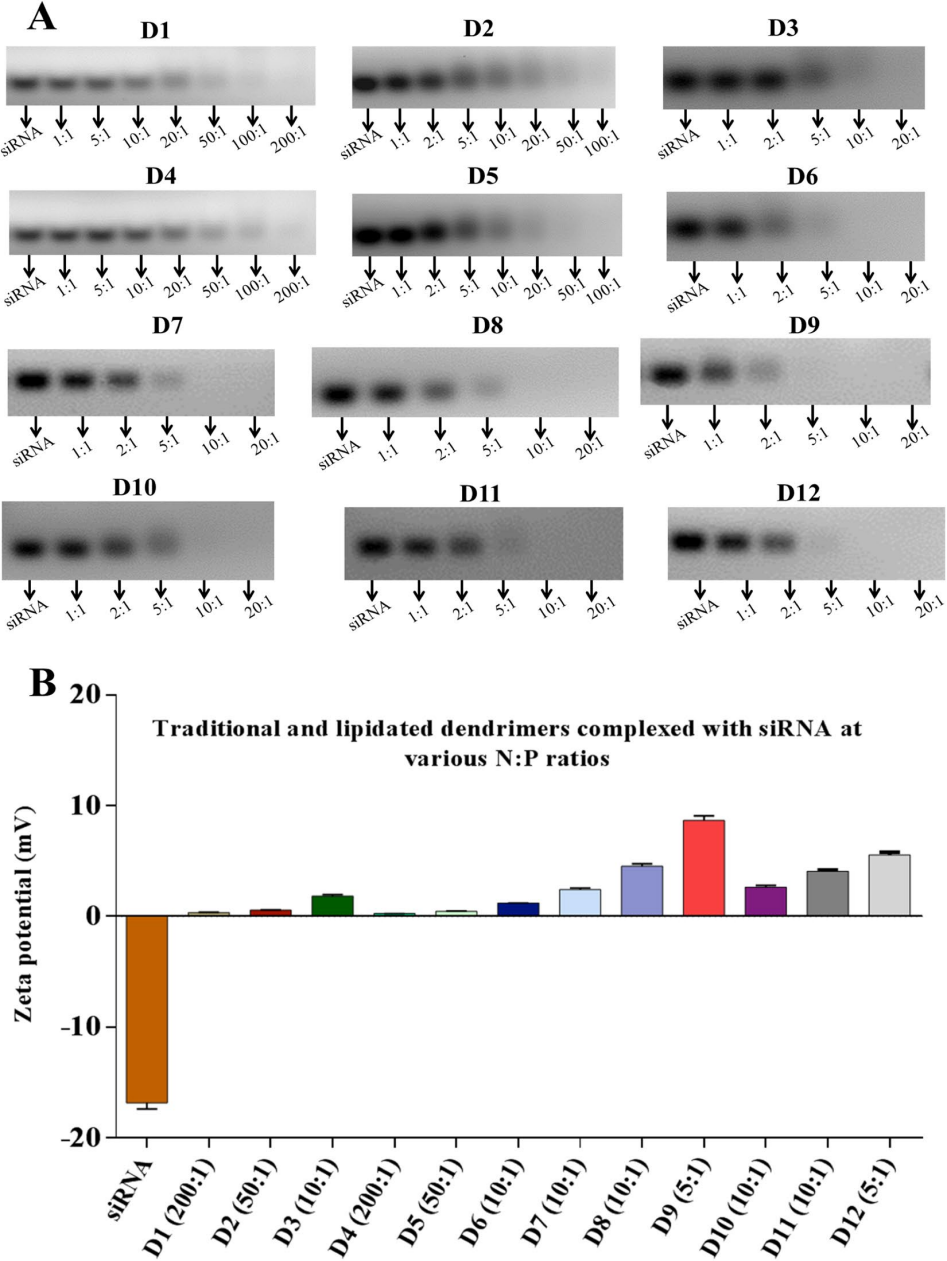
(**A**) *N* to *P* ratio for D1 to D6 (traditional) and D7 to D12 (lipidated) asymmetric peptide dendrimers with amine head group. (**B**) Net surface charge on siRNA and dendriplexes (at optimal *N* to *P* ratios) determined using zeta potential (mV) measurements.

All asymmetric peptide dendrimers demonstrated the ability to complex with siRNA across the *N* to *P* ratios trialled. The gel retardation assay results of traditional asymmetric peptide dendrimers (D1 to D6) are depicted in Fig. 4A, where those possessing a primary amine head group (D1 to D3) completely complex with siRNA (C1) at *N* to *P* ratios between 100:1 to 200:1 (D1, 4^+^), 20:1 to 50:1 (D2, 8^+^) and 5:1 to 10:1 (D3, 16^+^). Similar results were obtained for traditional asymmetric peptide dendrimers with the guanidine head groups (D4 to D6). The results indicate the complexation was charge dependent although the correlation was not linear, as 4^+^ (D1 and D4) asymmetric peptide dendrimers required higher *N* to *P* ratios compared to 8^+^ (D2 and D5), which was far less in the case of 16^+^ asymmetric peptide dendrimers (D3 and D6). This implies charge alone was not driving complexation of these systems, although it was the predominant factor.

Next, the lipidated asymmetric peptide dendrimers (D7 – D12) were tested for siRNA complexation ability. Compared to traditional asymmetric peptide dendrimers, side arm lipid (CA and DA) conjugated systems have an additional advantage of self-assembling into micelles in aqueous solution, which occurs at very low (μM) concentrations, commonly referred to as their CMC. Compared to traditional asymmetric peptide dendrimers, the siRNA complexation capabilities of lipidated asymmetric peptide dendrimers were found to be independent of charge (Fig. 4A). This may be attributed to the self-assembling nature of the lipidated dendrimers which can assist not only in charge based interaction with nucleic acids but also helps to encapsulate them. The lipidated asymmetric dendrimers form stable complexes with siRNA at far lower *N* to *P* ratios compared to native asymmetric peptide dendrimers, a case in point is the 4^+^ traditional asymmetric peptide dendrimers (D1 and D4), which require 100:1 and 200:1, while all 4^+^ lipidated asymmetric dendrimers (D7 and D10) fully complexed at a mere 5:1 to 10:1. The CA-conjugated asymmetric peptide dendrimers were shown to complex at *N* to *P* ratios of 10:1 in the case of D7 (4^+^) and D8 (8^+^), while D9 (16^+^) showed the lowest *N* to *P* ratio of 5:1 (Fig. 4A). The DA-conjugated asymmetric peptide dendrimers were shown to complex at *N* to *P* ratios of 10:1 in the case of D10 (4^+^) and D11 (8^+^), while D12 (16^+^), again showed the lowest *N* to *P* ratio of 5:1 (Fig. 4A).

The physicochemical characteristics of siRNA, namely high molecular weight, anionic charge and hydrophilicity prevent its passive diffusion across cellular membranes and into the cytosol, where it ultimately exerts its gene silencing effect^28^. Thus, effective delivery vectors are required to promote cytosolic delivery and gene silencing. Asymmetric peptide dendrimers due to their tunable cationic nature can effectively form complexes with siRNA and this was determined using a gel retardation assay. Documented evidence supports the role of dendriplexes, not only in protecting siRNA from nuclease degradation through effective compaction/condensation, but when the resultant dendriplexes retain a net positive charge, cellular internalization is assisted^29^. The gel retardation assay, in-conjunction with ethidium bromide allows the migration characteristics of nucleic acids (anionic) to be determined and visualized, while also enabling the influence of migration in the presence of asymmetric peptide dendrimers (cationic) to be determined. The fundamental driver of migration in this assay is influenced by the electrical current applied across a gel media. Ethidium bromide plays the role of an intercalator in the gel retardation assay, being used to detect uncomplexed siRNA as it intercalates into its native helical duplex. siRNA condensation in the presence of asymmetric peptide dendrimer disrupts this helical conformation, ejecting ethidium bromide from within the helical duplex as a result of compaction/condensation; this being observed by a reduction and eventually, complete loss of fluorescence in the agarose gel indicating comprehensive gene-condensation.

All asymmetric peptide dendrimers (D1 to D12) demonstrated the ability to complex siRNA albeit across a broad range of *N* to *P* ratios. The complexation ability of traditional asymmetric peptide dendrimers (D1 to D6) was found to be cationic charge dependent, but was not directly proportional to their generation/charge, indicating that charge alone was not driving complexation of these systems. The complexation ability (*N* to *P* ratio) of lipidated asymmetric peptide dendrimers (D7 to D12) was found to be consistently in the lower range, compared to traditional asymmetric peptide dendrimers. This may be attributed to the lipid components on the asymmetric peptide dendrimers, driving micellar formation in aqueous solution, a feature not observed with traditional asymmetric peptide dendrimers.

### siRNA-dendriplexes possess net positive charge

The net surface charge on siRNA-asymmetric peptide dendrimer complexes (dendriplexes) was determined at various *N* to *P* ratios, with a net positive charge considered important for interaction with the glycocalyx, the first step towards cellular internalization (Fig. 4B). Side arm lipidation on the dendrimers enhances the net surface positive charge of the dendriplexes essential which is for interaction with negatively charged cell membrane. For instance, D4 (4+) showed a net positive surface charge of 0.25 mV, compared to dendriplexes from lipidated asymmetric peptide dendrimers of the same charge D7 (4+) showed 2.64 mV. The lowest surface charge was observed with D4 (4^+^) asymmetric peptide dendrimer (+0.25 mV) while D9 (16^+^) exhibited the highest value at +9.0 mV.

The zeta potential measurements revealed a higher net positive charge on the lipidated compared to traditional dendriplexes required for interaction with the cell membrane and ultimately, cellular entry. Overall, the higher positive charge of dendriplexes formed with lipidated asymmetric peptide dendrimers compared to traditional asymmetric peptide dendrimers may be attributed to the micellar nature of these systems, where there is a higher concentration of charge expected.

### Asymmetric peptide dendrimers protect siRNA against RNase degradation

**Figure 5:**
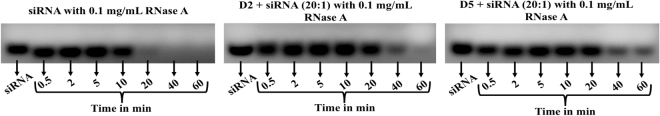
siRNA protection by D2 and D5 asymmetric peptide dendrimers.

The tremendous potential of siRNA-mediated target gene knockdown has been convincingly showcased across various preclinical models of the disease. However, the plasma half-life of native siRNA typically ranges from several minutes to 1 h, which limits their *in vivo* applicability. In an effort to address these shortfalls, we tested the ability of our asymmetric peptide dendrimers to protect siRNA in the presence of RNAse. These siRNA-asymmetric peptide dendrimer complexes were challenged for their stability in a nuclease rich (RNase A at 0.1 mg/mL) environment. Two different asymmetric dendrimers (8^+^ D2 and D5) with arg and lys head groups were complexed at 20:1 ratio (as per the gel retardation assay) and incubated with RNase A. Samples were aliquoted at different time points (30 s, 2, 5, 10, 20, 40 and 60 min) and analyzed using 2% agarose gel electrophoresis containing ethidium bromide. As shown in Fig. 5, naked siRNA duplex was stable in 0.1 mg/mL RNase A for up to 10 min. In contrast D2-siRNA dendriplexes were stable for 40 min while D5-siRNA dendriplexes were stable and visible up to 60 min in 0.1 mg/mL RNase A solution. This observation confirms the potential of our asymmetric peptide dendrimers in protecting their genetic cargo for extended periods (*c*.*f*. naked siRNA) in the presence of nucleases.

### Cytotoxicity profiling of asymmetric peptide dendrimers alone and siRNA-dendriplexes

A logical step towards considering asymmetric peptide dendrimers as safe vectors was to assess their cytotoxicity when applied alone, or when complexed with siRNA, via an MTS assay in H-4-II-E cells. The assay revealed that the entire asymmetric peptide dendrimer library when trialled either alone or as dendriplexes (using pre-optimized *N*:*P* ratios) are non-cytotoxic at the various concentrations trialled, when compared to Lipofectamine 2000, which is significantly cytotoxic (*p* < 0.00001).

**Figure 6:**
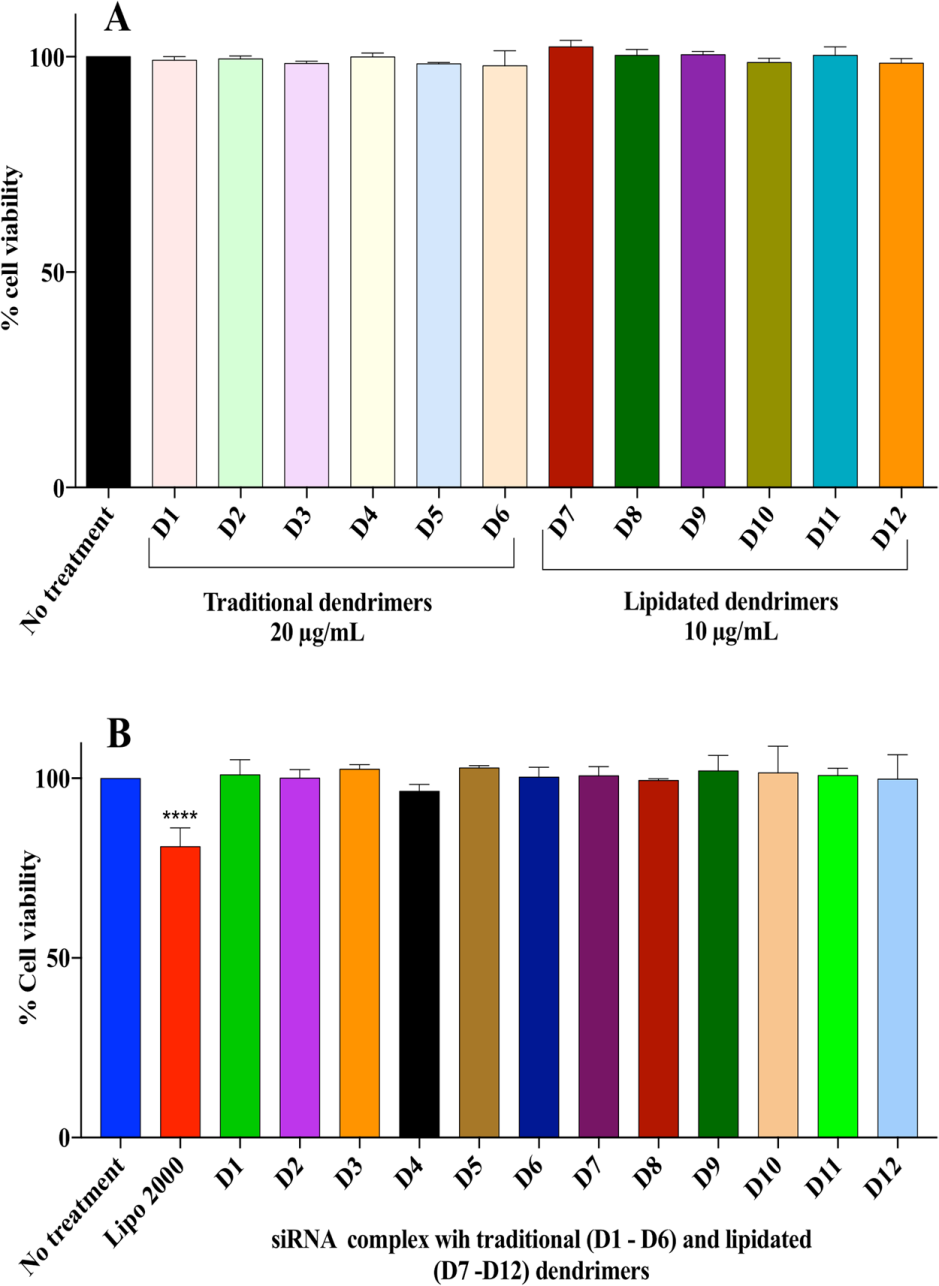
(**A**) Evaluation of cell viability on H-4-II-E cells after treatment with traditional and lipidated asymmetric peptide dendrimers. H-4-II-E cells were seeded at 1 × 10^4^ cells per well in 24-well plates and incubated at 37 °C in cell culture medium with 10% FBS for 24 h. Cells were exposed to FITC-siRNA (2 μg/mL), Lipofectamine 2000/FITC-siRNA (2 μg/mL) complex, or asymmetric peptide dendrimers/FITC-siRNA (2 μg/mL) complex in serum-free medium for 4 h. The complex was removed and cells incubated in 10% FBS medium for 24 h and imaged using fluorescence microscopy. Asymmetric peptide dendrimer internalization were visualized by representative bright field and fluorescence microscopy images of cells 24 h post treatment and compared to FITC-siRNA alone, Lipofectamine 2000/FITC-siRNA or Asymmetric peptide dendrimers/FITC-siRNA as indicated. Scale bar represents 200 μm. (**B**) Assessment of cell viability after exposure to traditional and lipidated asymmetric peptide dendrimers/siRNA complex H-4-II-E cells were exposed for 4 h to serum-free medium (no treatment), traditional (D1 to D6) and lipidated (D7 to D12) asymmetric peptide dendrimers /siRNA complex at various optimized N to P ratios, or to Lipofectamine 2000 /siRNA (0.1 pmol) complex. Cell viability was assessed after 4 h post treatment using the MTS assay. Results are expressed as percentage cell viability compared to untreated control cells and shown as mean ± S.E.M. (n=3 separate experiments), ****p<0.0001 using two way ANOVA.

To establish the cytotoxicity profile, H-4-II-E cells were first treated with native and lipidated asymmetric peptide dendrimers alone, with Lipofectamine 2000 serving as control. Both native and lipidated asymmetric peptide dendrimers (Fig. 6A) were non-toxic to cells, which led to us assessing their toxicity when complexed with siRNA next, at optimized *N* to *P* ratios (Fig. 6B). Compared to Lipofectamine 2000, all asymmetric peptide dendrimer-siRNA complexes were shown to be non-toxic with cell viabilities > 97% in all instances. In contrast, cell viability of Lipofectamine 2000, was significantly reduced by as much as 20% under identical incubation conditions.

### Internalization and delivery efficiency of cationic asymmetric peptide dendrimers using FITC-labelled siRNA

**Figure 7:**
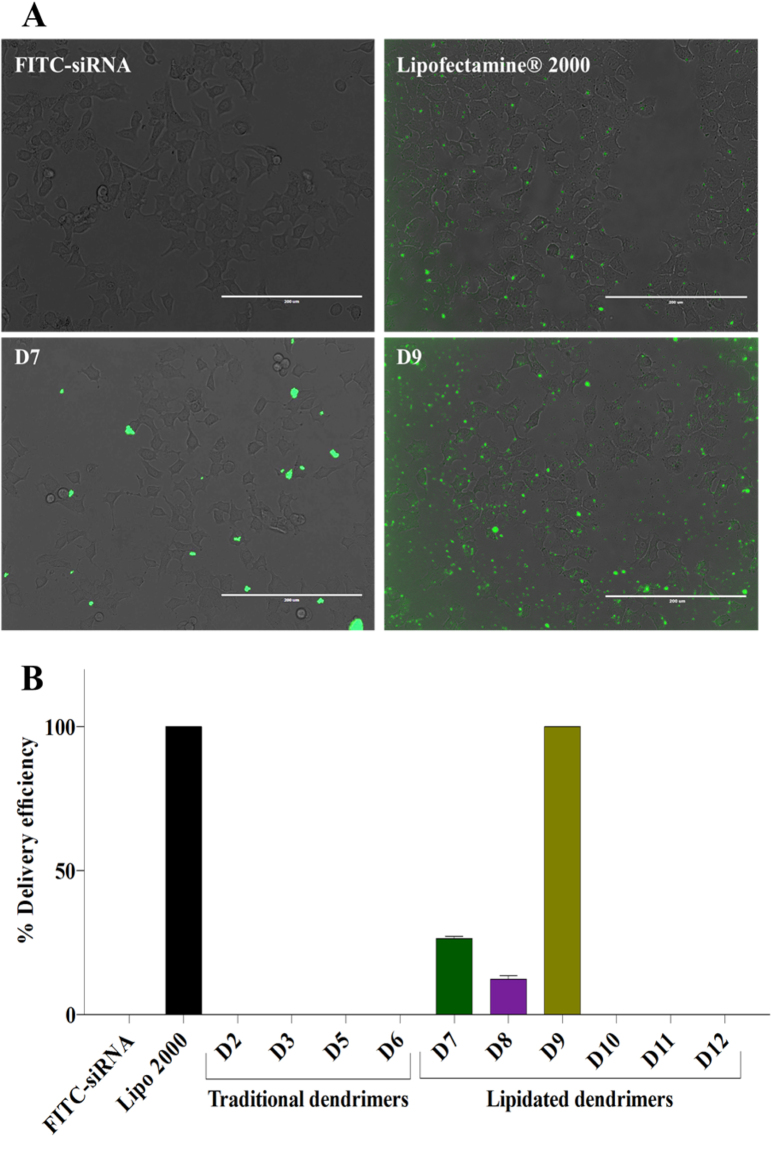
(**A**) Delivery efficiency of cationic asymmetric peptide dendrimers determined using FITC-labelled siRNA. H-4-II-E cells were seeded at 1 × 10^4^ cells per well in 24-well plates and incubated at 37 °C in cell culture medium with 10% FBS for 24 h. Cells were exposed to FITC-siRNA (2 μg/mL), Lipofectamine 2000/FITC-siRNA (2 μg/mL) complex, or asymmetric peptide dendrimers/FITC-siRNA (2 μg/mL) complex in serum-free medium for 4 h. The complex was removed and cells incubated in 10% FBS medium for 24 h and imaged using fluorescence microscopy. Asymmetric peptide dendrimer internalization were visualized by representative bright field and fluorescence microscopy images of cells 24 h post treatment and compared to FITC-siRNA alone, Lipofectamine 2000/FITC-siRNA or Asymmetric peptide dendrimers/FITC-siRNA as indicated. Scale bar represents 200 μm. (**B**) Asymmetric peptide dendrimers delivery efficiency using FITC-siRNA. The asymmetric peptide dendrimer internalization was reported as % FITC-siRNA delivery efficiency, compared to FITC-siRNA alone and Lipofectamine 2000/ FITC-siRNA, quantitated using Image J software.

The ability of traditional and lipidated cationic asymmetric peptide dendrimers to deliver nucleic acid to cells was evaluated next using FITC-labelled siRNA in H-4-II-E hepatocytes. All asymmetric peptide dendrimers (traditional and lipidated) were allowed to form a complex with FITC-labelled siRNA and their delivery efficiency was compared to Lipofectamine 2000 as a positive control, while FITC-siRNA alone served as the negative control. All cells were imaged at 24 h post treatment (Fig. 7A). FITC-siRNA alone did not exhibit any fluorescence, whereas lipidated asymmetric peptide dendrimers D7 (4^+^ CA) and D9 (16^+^ CA) were successful in delivering FITC-siRNA to the cells (Fig. 7A). As shown in Fig. 7B, D7 and D8 exhibited up to 28% delivery efficiency, while in the case of Lipofectamine 2000 and D9 delivery efficiency was near quantitative. This assay was performed merely to assess the internalization potential of our asymmetric peptide dendrimers library and thus, quantification of the data in terms of fluorescence intensity was not assessed here. These results indicate that the lipidated asymmetric peptide dendrimers (particularly D9) show promise as potential non-cytotoxic, efficient siRNA delivery vectors.

**Figure 8:**
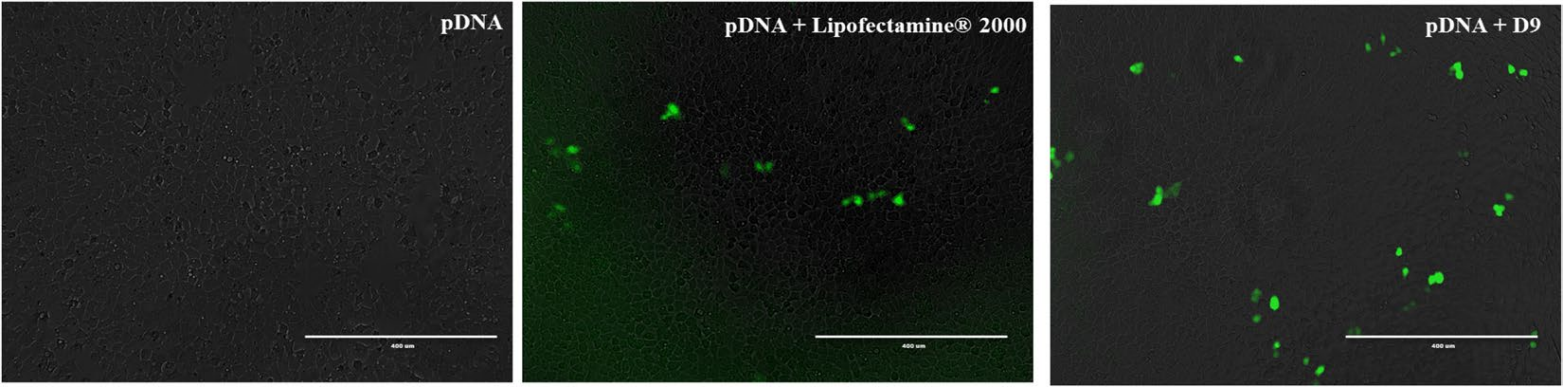
Delivery efficiency of cationic asymmetric peptide dendrimer D9 determined using pEGFP expression. H-4-II-E cells were seeded at 1 × 10^4^ cells per well in 24-well plates and incubated at 37 °C in cell culture medium with 10% FBS for 24 h. Cells were exposed to pEGFP (1 μg/mL), Lipofectamine 2000/pEGFP (1 μg/mL) complex, or asymmetric peptide dendrimers/pEGFP (1 μg/mL) complex in serum-free medium for 4 h. The complex was removed and cells incubated in 10% FBS medium for 48 h and imaged using fluorescence microscopy. pEGFP expressions were visualized by representative bright field and fluorescence microscopy images of cells 48 h post treatment and compared to pEGFP, Lipofectamine 2000/pEGFP or asymmetric peptide dendrimers/pEGFP as indicated. Scale bar represents 400 μm.

Next, all asymmetric peptide dendrimers (native and lipidated) were allowed to form complexes with pEGFP and their delivery efficiency was compared to Lipofectamine 2000 as a positive control, while naked pEGFP served as a control. All cells were imaged at 48 h post treatment (Fig. 8). As expected, the naked pEGFP failed to fluoresce, whereas lipidated asymmetric peptide dendrimer D9 (16^+^ CA) successfully delivered pEGFP *in vitro* (Fig. 8). This assay was performed merely to assess the internalization potential of our asymmetric peptide dendrimer library and quantitation of fluorescence intensity was not deemed necessary. These results further corroborate that the lipidated asymmetric peptide dendrimers (D9) display potential as a non-cytotoxic transfecting carriers for genetic cargo of varied architecture. All other peptide dendrimers failed to deliver pEGFP into the cells. This may be due to their lower net positive charge (density) or lower pay load capacity.

### Internalization and delivery efficiency of cationic asymmetric peptide dendrimers using FAM-ssDNA in myotubes

**Figure 9:**
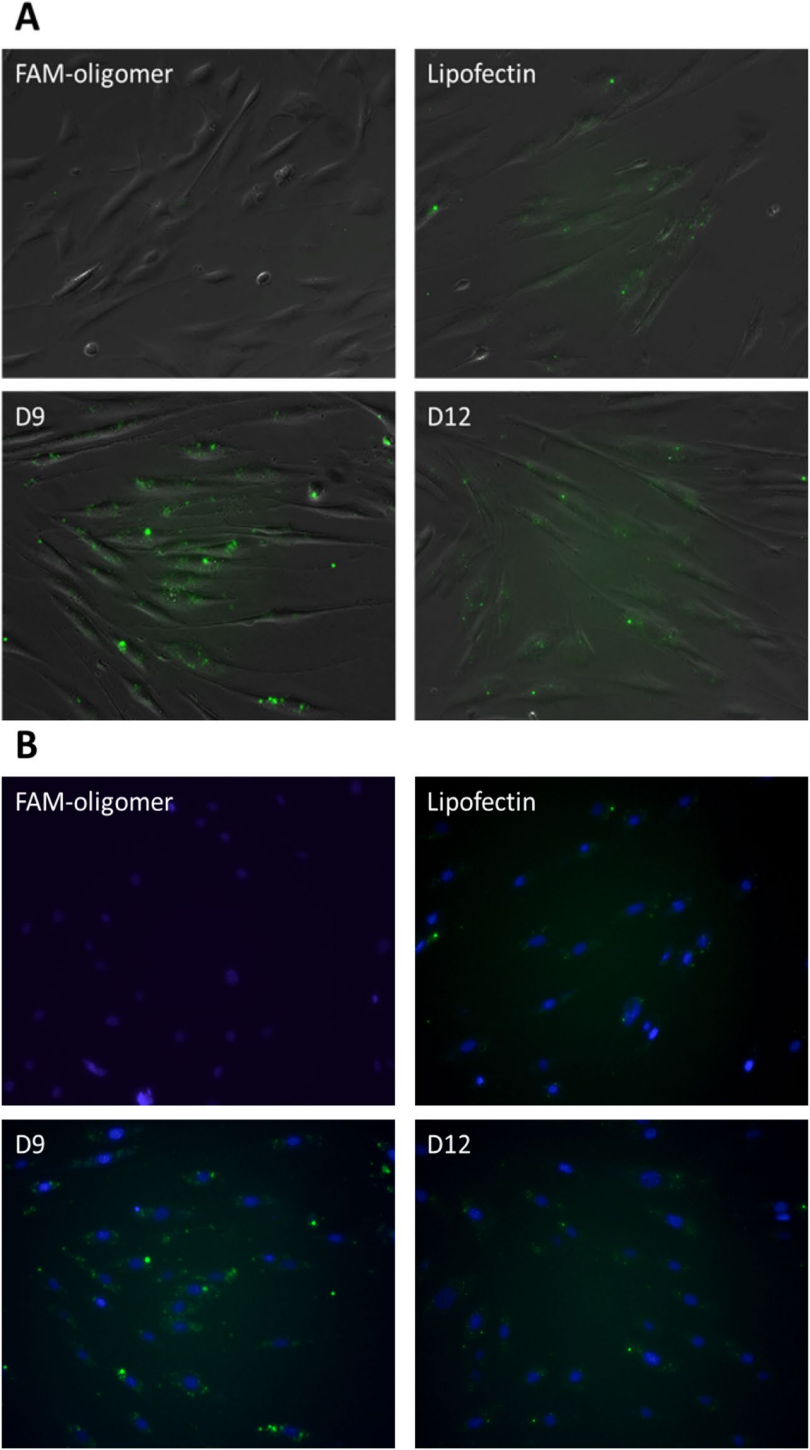
Delivery efficiency of asymmetric peptide dendrimers determined using FAM-ssDNA in H2K mdx cell line. DAOY cells were plated in a 24-well plate 24 hours prior to transfection at 2.5 × 10^4^ cells/well. Cells were transfected with FAM-ssDNA (3.5 μg/mL), Lipofectin®/ FAM-ssDNA (3.5 μg/mL) complex, or asymmetric peptide dendrimer/FAM-ssDNA (3.5 μg/mL) complex in serum-free medium for 24 h. Twenty-four hours after transfection, the cell nucleus were stained with Hoechst for 15 minutes, washed with PBS before asymmetric peptide dendrimers internalization were visualized by representative in (**A**) bright field and fluorescence microscopy; and (**B**) UV and fluorescein-based microscopy with nucleus staining.

All asymmetric peptide dendrimers (native and lipidated) were next allowed to form complexes with FAM-labelled ssDNA and their delivery efficiency was compared to Lipofectin^®^ as a positive control, while naked FAM-ssDNA served as a control. All cells were imaged at 24 h post treatment (Fig. 9). As expected, the naked FAM-ssDNA failed to fluoresce, whereas D9 (16^+^ CA) and D12 (16^+^ DA) successfully delivered FAM-ssDNA *in vitro* (Fig. 9). And again, this assay was performed merely to assess the internalization potential of our asymmetric peptide dendrimer library and quantitation of fluorescence intensity was not deemed necessary. These results further corroborate that the lipidated asymmetric peptide dendrimers (particularly D9) displays potential as a non-cytotoxic transfecting carrier for double and single stranded gene-based cargo.

### Internalization and delivery efficiency of cationic asymmetric peptide dendrimers using FAM-ssDNA in DAOY

**Figure 10:**
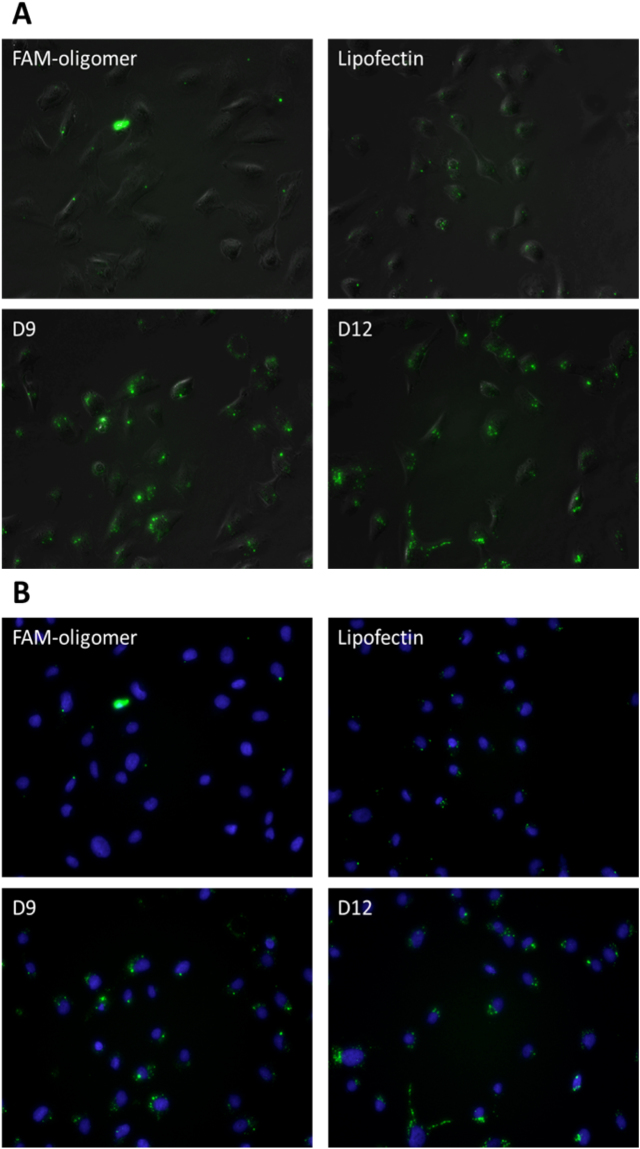
Delivery efficiency of asymmetric peptide dendrimers determined using FAM-ssDNA in DAOY cell line. DAOY cells were plated in a 24-well plate 24 hours prior to transfection at 2.5 × 10^4^ cells/well. Cells were transfected with FAM-ssDNA (3.5 μg/mL), Lipofectin®/ FAM-ssDNA (3.5 μg/mL) complex, or asymmetric peptide dendrimer/FAM-ssDNA (3.5 μg/mL) complex in serum-free medium for 24 h. Twenty-four hours after transfection, the cell nucleus were stained with Hoechst for 15 minutes, washed with PBS before asymmetric peptide dendrimers internalization were visualized by representative in (**A**) bright field and fluorescence microscopy; and (**B**) UV and fluorescein-based microscopy with nucleus staining.

All asymmetric peptide dendrimers (traditional and lipidated) were also assessed for gene delivery capabilities in DAOY cells, which are traditionally considered to be difficult to transfect cells. Asymmetric peptide dendrimers were complexed with FAM-ssDNA and their delivery efficiency was compared to Lipofectin^®^ as a positive control, while naked FAM-ssDNA served as the negative control. All cells were imaged at 24 h post treatment (Fig. 8). As expected, naked FAM-ssDNA failed to fluoresce, whereas lipidated asymmetric peptide dendrimers D9 (16^+^ CA) and D12 (16^+^ DA) were shown to deliver FAM-ssDNA efficiently to DAOY cells (Fig. 10).

The observed delivery efficiencies of ssDNA, siRNA and pEGFP measured across a range of cell types (liver, muscle and brain) clearly demonstrates the versatility of CA-conjugated asymmetric peptide dendrimer (16^+^; D9) as possessing both far superior delivery capabilities when compared to Lipofectamine 2000 or Lipofectin in rat hepatocytes, mouse myoblast and human medulloblastoma, while being comprehensively non-cytotoxic. The DA-conjugated asymmetric peptide dendrimer (16 + ; D12) was also found to be effective with muscle and brain cells, while failed to deliver the cargo to liver cells. This information can be used to optimize the vectors for target specific gene delivery. The superior delivery efficiency of lipidated dendrimers (D9 and D12) can be attributed to the self-assembling nature of these asymmetric peptide dendrimers, which occurs at very low (µM) concentrations. Further evidence is that none of the traditional asymmetric peptide dendrimers were shown to deliver the nucleic acids to the array of cell types trialled. This may be attributed to the structurally much smaller nucleic acid-asymmetric peptide dendrimer complexes possessing a lower net positive charge density, which in turn reduces their payload capacity and inability to effectively complex with the gene-based cargo.

## Conclusion

Successful targeted delivery and efficient gene transfection outcomes is a complex and challenging feat, most often requiring a suitable vehicle with a high capacity for nucleic acid loading and one that ensures its efficient and timely release in the nucleus or cytosol. In addition to systemic challenges, inefficient delivery vectors have compromised therapeutic efficacy and ultimately, the potency of siRNA-based gene therapy. To address the limitations of commercially available high generation cationic dendrimers, natural amino acid-based asymmetric peptide dendrimers were designed, synthesized and characterized in this study. Such asymmetric peptide dendrimers are emerging as a highly versatile, biocompatible vector class, and one that is expected to overcome the many challenges that have plagued traditional chemical carriers, such as poor rates of delivery/transfection efficiencies and their appreciable cytotoxicity. Further and more crucial, the asymmetric nature of these dendrimer systems allows side arm conjugation of targeting ligands without masking their cationic charge. We propose that nucleic acid-based therapeutics when combined with appropriately functionalized asymmetric peptide dendrimers as highlighted in this study, will create the next generation of safe, effective and biocompatible gene-based treatment strategies.

## Materials and Methods

### Materials

All chemicals were commercially sourced and used without further purification. Fluorenylmethyl-oxycarbonyl (Fmoc) amino acids (L-isomer) and Rink amide resin (200–400 mesh) were purchased from NovaBiochem (Australia) and Chem-impex International, Inc (USA). The solvent used throughout the synthesis was peptide grade *N*,*N*-Dimethylformamide (DMF; Merck). Trifluoroacetic acid (TFA), *O*-Benzotriazole-*N*,*N*,*N′*,*N′*-tetramethyl-uronium-hexafluoro-phosphate (HBTU), *N*,*N-*diisopropylethylamine (DIPEA), Dichloromethane (DCM), Triisopropylsilane (TIPS), piperidine, 2-Mercapthoethanol, ammonium persulfate (APS), Dulbecco’s modified Eagle’s medium (DMEM), Fetal bovine serum (FBS), Horse serum (HS), Accutase^®^ solution, cholic and decanoic acid, Poly-D-Lysine were purchased from Sigma-Aldrich (Australia). Eagle’s Minimum Essential Medium (EMEM) was purchased from ATCC (supplied by *In vitro* Technologies, Australia). Penicillin/streptomycin solution, Lipofectamine 2000, Lipofectin, Dulbecco’s phosphate-buffered saline without Calcium or magnesium (PBS), GlutaMAX™ (100×) were purchased from Life Technologies (Australia). FITC-conjugated control siRNA (sc-36869) was purchased from Santa Cruz Biotechnology Inc., CA., USA. Fluorescein labelled single-stranded DNA for monitoring uptake in DAOY cells and *mdx* mice myotubes was purchased from Integrated DNA Technology Inc (Iowa, USA). Matrigel was purchased from Corning Inc., New York, USA. Chicken embryo extract was purchased from *In Vitro* Technologies (Australia).

### Cationic asymmetric peptide dendrimer synthesis

The library of low-generation asymmetric peptide dendrimers (D1 to D6, Fig. 2, S1 Fig) varied in charge density (as 4^+^, 8^+^ and 16^+^) and head group (lysine or arginine) chemistry, being designed and synthesized using well-established Fmoc-SPPS strategies^6^. With the aim of enhancing nucleic acid delivery *in vitro*, a subset of asymmetric peptide dendrimers were prepared with side arm functionalization using either CA or DA (D7 to D12, Fig. 2, S1 Fig). All the asymmetric peptide dendrimers were synthesized on an insoluble solid support (rink amide resin, 0.47–0.79 mmol/g loading) with sequential addition of pre-activated (0.5 M HBTU in DMF and DIPEA) amino acid until the desired asymmetric peptide dendrimer had been constructed. Each coupling step was monitored for free amine using the ‘*Ninhydrin test*’^30^ and absorbance was measured at 570 nm using UV spectrophotometry (Varian Cary 50 UV-Vis). Cleavage of the target asymmetric peptide dendrimer off-resin was performed under acidic conditions using a TFA mixture (TFA/TIPS/H_2_O/DCM – 90:2.5:2.5:5 *v/v*). To enable the site specific attachment of lipids to asymmetric dendrimers, the amino acid building block Fmoc-Lys(Dde)-OH was synthesized and characterized by ESI ± MS. The precise architecture of asymmetric dendrimers allowed site specific conjugation of various lipids (CA or DA) to the side arm ensuring the cationic head groups remain available for nucleic acid complexation.

All asymmetric peptide dendrimers (100 mg crude) were purified using preparative RP-HPLC on a Waters system (USA, Model 600 controller, 2996 photodiode array detector, Elite Alltech degassing system and MassLynx^TM^ software) with C_18_ column (Grace Vydac; particle size 10 µm pore size, id = 22 mm × 250 mm). Asymmetric peptide dendrimer purity was assessed via an analytical RP-HPLC system (Shimadzu (Japan) controller-CBM-20A, pump A-LC-10AD, autosampler – SIL-10AXL, a variable wavelength UV/vis detector, degasser – DGU As Prominance) with a C_18_ column (Grace Vydac; particle size 5 µm pore size, id = 4.6 mm × 250 mm, pore size: 300 Å). The detection wavelength in RP-HPLC and analytical RP-HPLC was 219 nm. The mobile phase employed was: Solvent A; (100% H_2_O), solvent B; (90% CH_3_CN_(aq)_), with a flow rate of 10 mL/min used for preparative RP-HPLC. In the case of analytical RP-HPLC, mobile phase employed was: Solvent A; (0.1% v/v TFA in H_2_O), Solvent B; (0.1% v/v TFA CH_3_CN_(aq)_). Conditions, linear gradient from 0–100% B over 20 min, flow rate of 1 mL/min at 25 °C. Mass spectrometric analysis (ESI ± MS and HR-MS) was performed on an Applied Biosystem/MDS Sciex Q-TOF LC/MS/MS system (Agilent technologies, Australia) and Accurate-Mass Q-TOF LC/MS (Agilent technologies, Australia) systems.

### Critical micelle concentration determination

To determine the critical micelle concentration (CMC) of lipidated asymmetric peptide dendrimers, dynamic light scattering technique^26^ employing a Malvern Zetasizer, NANO ZS (Malvern Instruments Limited, U.K.), equipped with a 4 mW He–Ne laser operating at a 633 nm, was used. The scattered light was detected at an angle of 173°, using an optical arrangement known as non-invasive back scatter (NIBS), which maximizes the detection of scattered light while maintaining signal quality. This provides the exceptional sensitivity that is required for measuring the size of entities such as nanoparticles and polymer micelles, at low concentrations. Measurements were carried out in a polystyrene cell at 25 °C. A series of peptide dendrimer solutions ranging from 50 nM to 200 µM were prepared from an aqueous stock solution. Representative asymmetric peptide dendrimers (D7, D9) were analyzed using transmission electron microscopy (TEM) to determine their morphology and self-assembling properties. Briefly, the copper grids were first dipped into the sample solution and immediately transferred to liquid nitrogen for freezing (10 min). Later, the copper grid was freeze dried and analyzed using TEM (JEOL 1010).

### Gel retardation assay for optimal *N* (dendrimer): *P* (gene) ratio determination

To confirm the ability of our asymmetric peptide dendrimers to form stable complexes with siRNA, a gel retardation assay was developed and optimized^27^. Asymmetric peptide dendrimer (1 mg/mL)-siRNA (10 μM, 10 μL) complexes were prepared at different *N* (amine) to *P* (phosphate) ratios (i.e. from 1:1 to 200:1) based on the molar ratios of phosphates (from siRNA) and cationic charge present on the dendrimers. Complexes with equal amounts of siRNA were then loaded on 2% agarose gels with glycerol-based loading buffer (1 μL). Electrophoresis was performed in Tris/Acetate/EDTA (TAE) buffer at 80 V for 30 min with siRNA bands visualized using ethidium bromide, where visible bands indicated incomplete complexation.

### Asymmetric peptide dendrimer-siRNA complex stability to RNase A

To assess the ability of our asymmetric peptide dendrimers to protect siRNA from ribonuclease degradation a RNase protection assay was performed. Asymmetric peptide dendrimer (D2 and D5) (1 mg/mL)-siRNA (40 μM, 10 μL) complexes were prepared at 20:1 (*N* to *P* ratio) based on the gel retardation assay. siRNA-asymmetric peptide dendrimer complexes were then incubated in 0.1 mg/mL RNase A solution at 37 °C with siRNA serving as a control. An aliquot (4 μL) of sample was collected at different time intervals (30 s, 2, 5, 10, 20, 40 and 60 min). The aliquoted samples were mixed with 1% SDS to inactivate RNase A and kept on ice. Samples were then loaded on 2% agarose gels with glycerol-based loading buffer (1 μL). Electrophoresis was performed in Tris/Acetate/EDTA (TAE) buffer at 80 V for 30 min with siRNA bands visualized using ethidium bromide, where visible bands indicated ribonuclease stability.

### Zeta potential measurement

The zeta potential of asymmetric peptide dendrimer and asymmetric peptide dendrimer-siRNA complexes were measured with a Malvern Zetasizer, NANO ZS (Malvern Instruments Limited, U.K.) using a disposable capillary cell (DTS1070)^31^. Asymmetric peptide dendrimer solution at 1 mg/mL concentration or the siRNA-asymmetric peptide dendrimer complexes at optimized *N* to *P* ratios were diluted to 750 μL using 10 mM NaCl and measurements performed in a zeta cell (Malvern Instruments, DTS1060) at 25 °C with an applied voltage of 150 V.

### Cell culture and treatment

The rat (*Rattus norvegicus*) hepatoma-derived H-4-II-E hepatocyte cell line^32^ was obtained from American Type Culture Collection (ATCC^®^ CRL1548™) and grown in DMEM medium supplemented with 10% (v/v) FBS, 100 IU/mL penicillin, 100 µg/mL streptomycin with GlutaMAX™ (1% v/v), at 37 °C in a humidified atmosphere with 5% CO_2_.

The mouse H-2Kb-tsA58 (H2K) mdx myoblast^33^ was cultured as described by Mann *et al*.^34^. Briefly, the cells were cultured in Matrigel-coated flasks (100 μg/ml) at 33 °C, and 10% CO_2_ in DMEM, 20% FBS, 10% horse serum, 0.5% Chicken embryo extract.

The human medulloblastoma cell line (DAOY)^35^ was obtained from American Type Culture Collection (ATCC^®^ HTB-186™) and cultured in EMEM medium supplemented with 10% FBS at 37 °C, 5% CO_2_ under humidified conditions.

### *In vitro* cytotoxicity profiling using the MTS assay

Cytotoxicity profiling of our asymmetric peptide dendrimer library alone, or when complexed with siRNA was performed using an MTS assay^36^. H-4-II-E cells were seeded into 96-well plates at a density of 7,500 cells per well (100 µL). After 24 h, cells were treated with either of the following: serum free media, asymmetric peptide dendrimers at 20 µg/mL (traditional) or 10 µg/mL (lipidated), siRNA-dendrimer complexes at various *N* to *P* ratios determined from the gel retardation assay. Cells were incubated at 37 °C for 4 h, with Lipofectamine 2000 serving as control. Complexes were then removed and MTS assay was performed as described below.

For the MTS assay, the CellTiter 96^®^ Aqueous One Solution Cell Proliferation Assay kit (Promega, Australia) was used following the manufacturer’s instructions. Briefly, 50 µL of the MTS reagent was added into each well and cells were incubated at 37 °C for 3 h, after which the absorbance was detected at 490 nm with a microplate reader (Biotek Synergy H4). Each experiment was performed in triplicate.

### Delivery efficiency of asymmetric peptide dendrimers in H-4-II-E cells

Delivery efficiency of select, asymmetric peptide dendrimers in H-4-II-E cells was determined using FITC-labelled siRNA. Lipofectamine 2000, a commercially available transfecting agent was used as a standard, to compare the delivery efficiency of FITC-labelled siRNA with synthesized asymmetric peptide dendrimers. H-4-II-E cells were seeded at a density of 1 × 10^4^ cells per well in 24 well plates and incubated for 24 h. FITC-labelled siRNA was transfected in H-4-II-E cells using Lipofectamine 2000 (as per manufacturer’s protocol) *vs* asymmetric peptide dendrimers at concentrations derived from earlier optimized *N* to *P* ratios from gel retardation studies. After 4 h of treatment, the complexes were removed, and the cells incubated at 37 °C overnight with culture media. The green fluorescence of FITC-labelled siRNA was observed and imaged after 24 h post treatment using EVOS^®^ digital inverted fluorescence microscopy. The number of fluorescent cells was counted in five different fields using Image J software, and results were expressed as the percentage delivery efficiency. Each experiment was performed in triplicate.

For pEGFP plasmid transfection studies, H-4-II-E cells were seeded at a density of 1 × 10^4^ cells per well in 24 well plates and incubated for 24 h. Cells were then incubated with the asymmetric peptide dendrimer/DNA complexes (50 μg dendrimer and 1 μg pEGFP plasmid) or with Lipofectamine 2000/DNA complexes for 4 h. The GFP expressing live cells were then observed at 48 h post transfection using EVOS^®^ digital inverted fluorescence microscopy.

#### Myotubes

To prepare for transfection, myoblast cells were differentiated into myotubes by plating into a 24-well plate coated with 100 μg/mL Matrigel followed by 50 μg/mL poly-D-lysine at 2.5 × 10^4^ cells/well in DMEM 5% HS. The plate was then incubated at 37 °C, 5% CO_2_ for ≈48 hrs to allow the cells to differentiate into myotubes. The myotubes were then transfected with a fluorescein (FAM)-ssDNA oligomer using Lipofectin^®^ as standard transfection reagent (following manufacturer’s protocol), in comparison with asymmetric peptide dendrimers and naked FAM-ssDNA. Twenty-four hours after transfection, cell nuclei were stained with Hoechst for 15 minutes, washed with PBS before being observed with Olympus TS-100 inverted fluorescence microscopy.

#### DAOY cells

DAOY cells were plated in a 24-well plate at 2.5 × 10^4^ cells/well, 24 hours prior to transfection. Cells were then transfected with a fluorescein (FAM)-ssDNA oligomer using Lipofectin^®^ as standard transfection reagent (following manufacturer’s protocol), in comparison with asymmetric peptide dendrimers and naked FAM-ssDNA. Twenty-four hours after transfection, cell nuclei were stained with Hoechst for 15 minutes, washed with PBS before being observed with Olympus TS-100 inverted fluorescence microscopy.

### Statistical analysis

All experiments were repeated three times independently. Results were reported as mean values ± standard error of mean (SEM). Data was analyzed using Prism 6.0 software (GraphPad software, Inc., USA). Differences were considered statistically significant when *p < *0.05.

## Electronic supplementary material

Supplementary Information
