# Supplementary material for: Self-assembling asymmetric peptide-dendrimer micelles – a platform for effective and versatile in vitro nucleic acid delivery
Source: Sci Rep. 2018 Mar 19;8:4832. doi: 10.1038/s41598-018-22902-9 (PMC5859181; doi:10.1038/s41598-018-22902-9)
Supplement: Supplementary file 1 — Supplementary Information [file 41598_2018_22902_MOESM1_ESM.pdf]

Self-assembling asymmetric peptide-dendrimer micelles – a platform for effective and versatile *in vitro* nucleic acid delivery

Ganesh R. Kokil[1], Rakesh N. Veedu[2,3,4*], Bao Tri Le[2,3], Grant A. Ramm[5,6*], and Harendra S. Parekh[1*]

[1]School of Pharmacy, Pharmacy Australia Centre of Excellence, The University of Queensland, Brisbane, QLD, Australia.

[2]Center for Comparative Genomics, Murdoch University, Murdoch, WA, Australia.

[3]Western Australian Neuroscience Research Institute, Perth, WA, Australia.

[4]School of Chemistry and Molecular Biosciences, The University of Queensland, Brisbane QLD, Australia.

[5]The Hepatic Fibrosis Group, QIMR Berghofer Medical Research Institute, Brisbane, QLD, Australia.

[6]Faculty of Medicine and Biomedical Sciences, The University of Queensland, Brisbane, QLD, Australia.

R.Veedu@murdoch.edu.au

Grant.Ramm@qimrberghofer.edu.au

Correspondence to h.parekh@uq.edu.au

Supplementary figure 1: Chemical structures and mass spectrum of asymmetric peptide dendrimers

D1

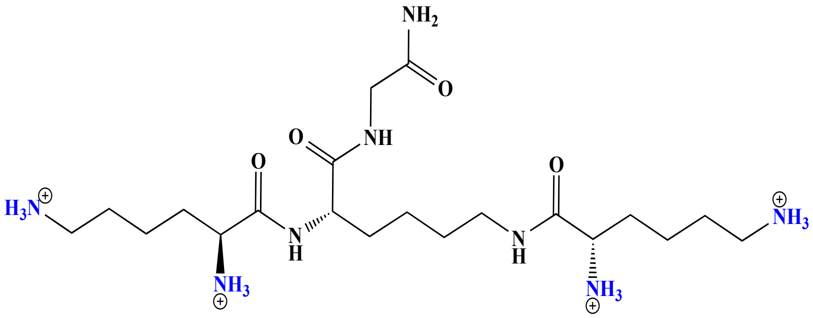

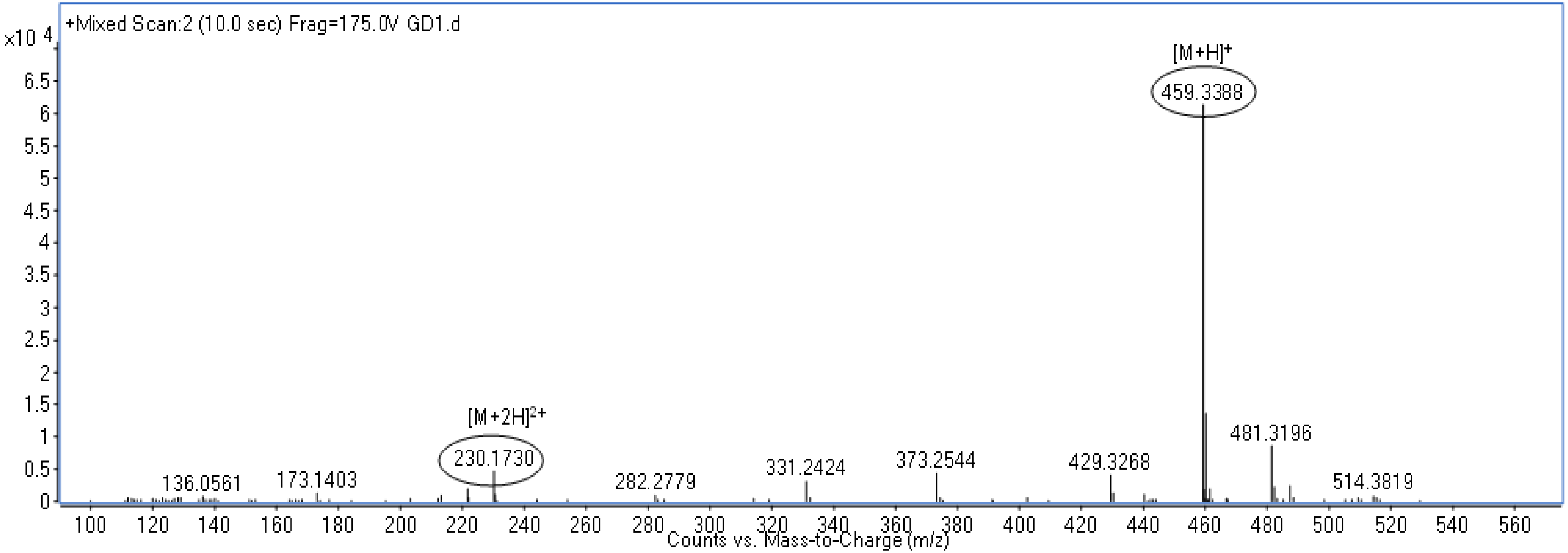

D2

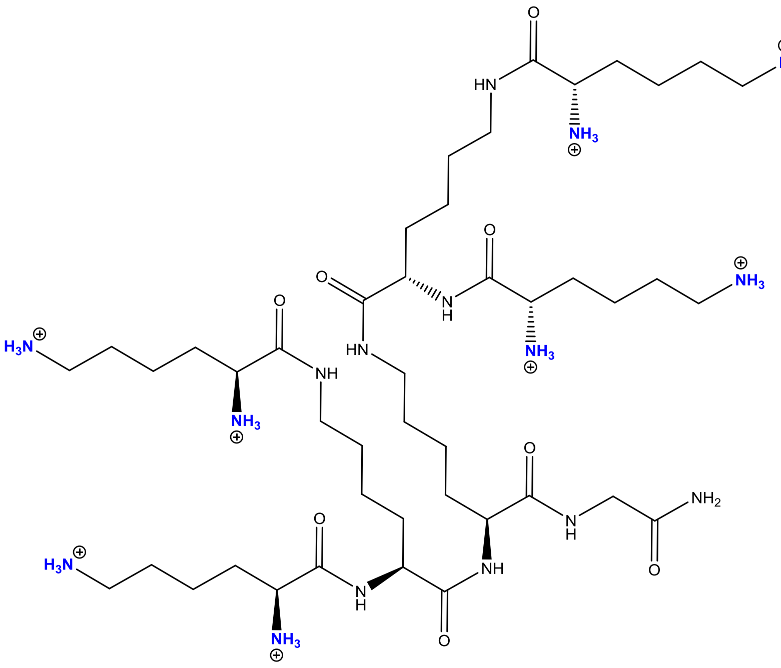

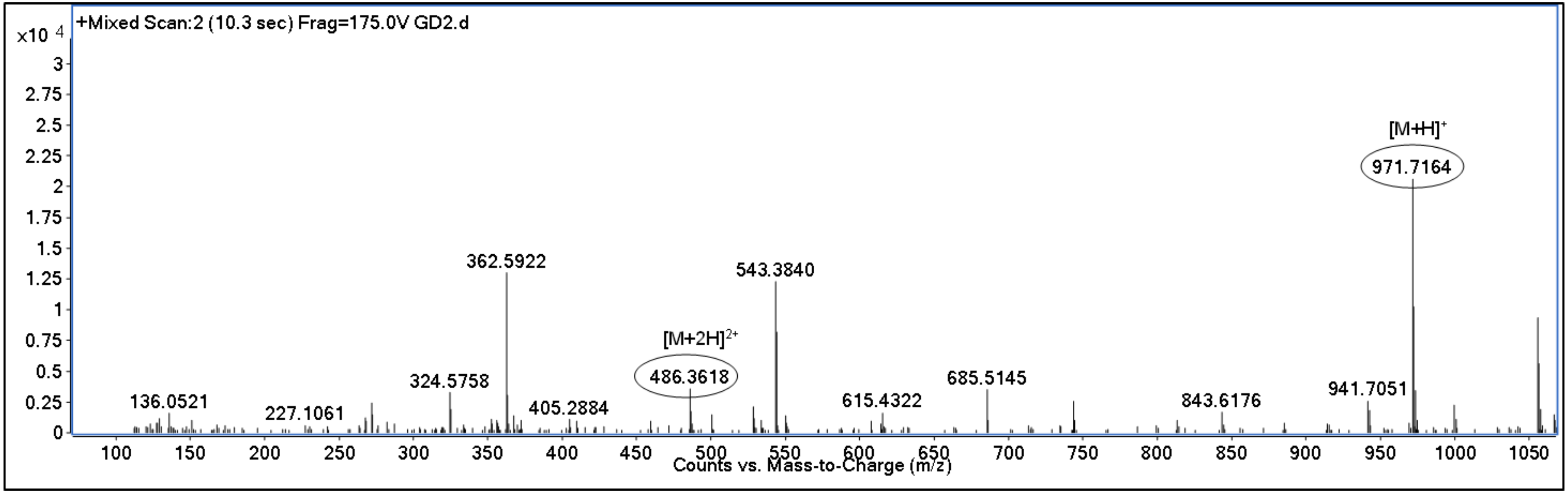
+Mixed Scan:2 (10.3 sec) Frag=175.0V GD2.d
x10 4
[M+H]+
971.7164
362.5922
543.3840
[M+2H]2+
486.3618
136.0521
227.1061
324.5758
405.2884
615.4322
685.5145
843.6176
941.7051
Counts vs. Mass-to-Charge (m/z)

D3

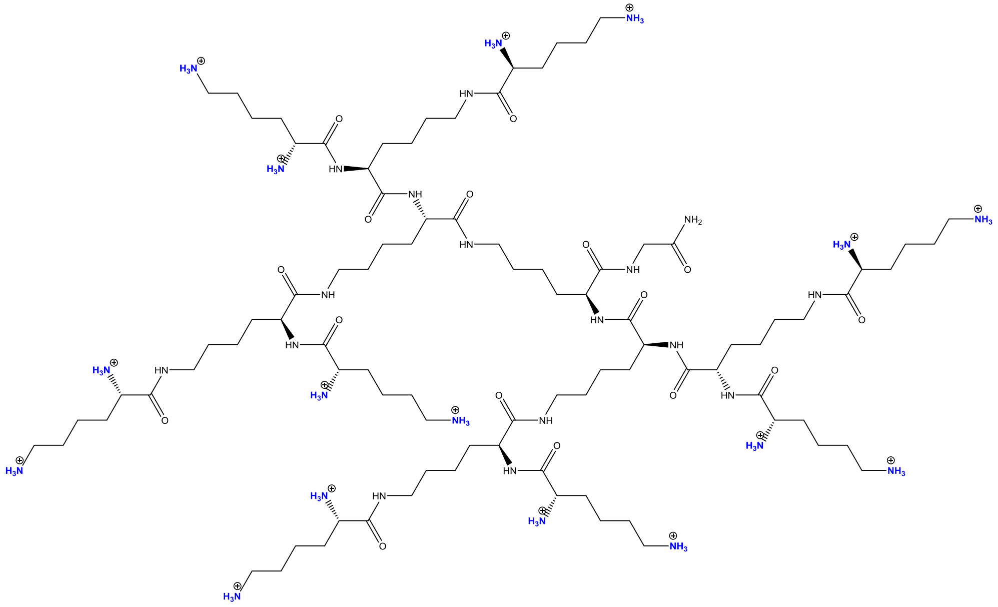

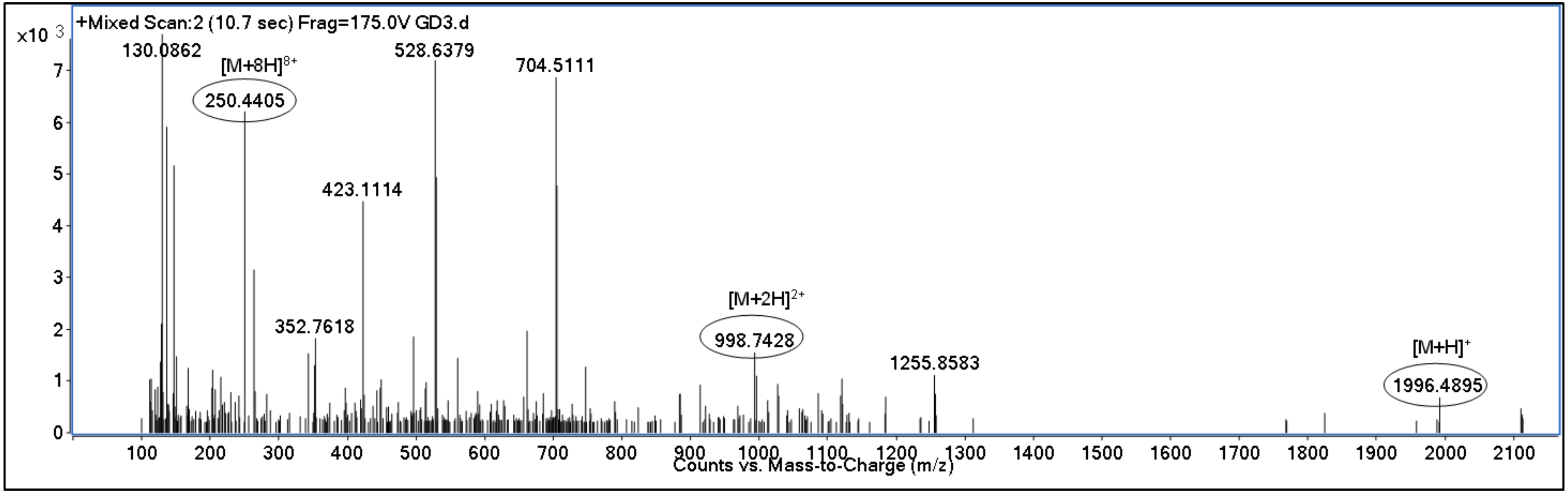

+Mixed Scan:2 (10.7 sec) Frag=175.0V GD3.d
x10 3
130.0862
[M+8H]8+
250.4405
528.6379
704.5111
423.1114
352.7618
[M+2H]2+
998.7428
1255.8583
[M+H]+
1996.4895
Counts vs. Mass-to-Charge (m/z)

D4

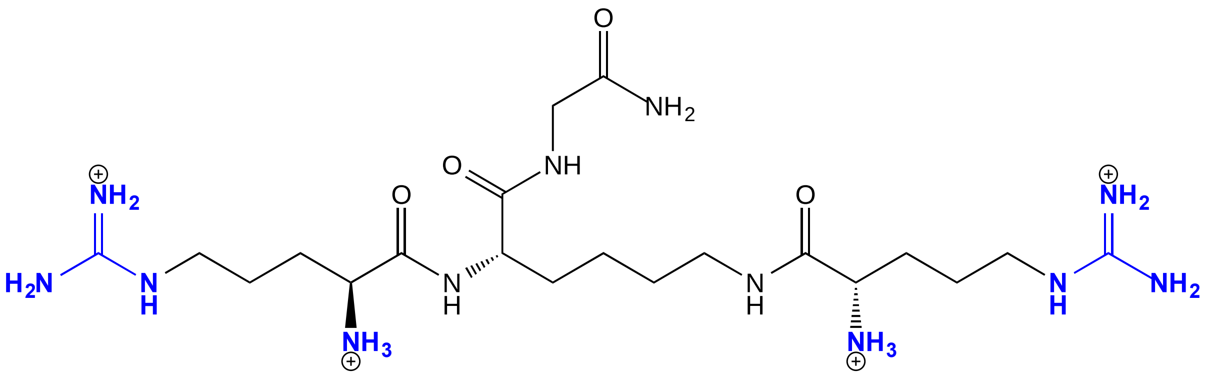

O
NH_2
NH
O
O
O
NH_2
NH_2
H_2N
N
H
N
H
N
H
N
H
NH_2
NH_3
NH_3

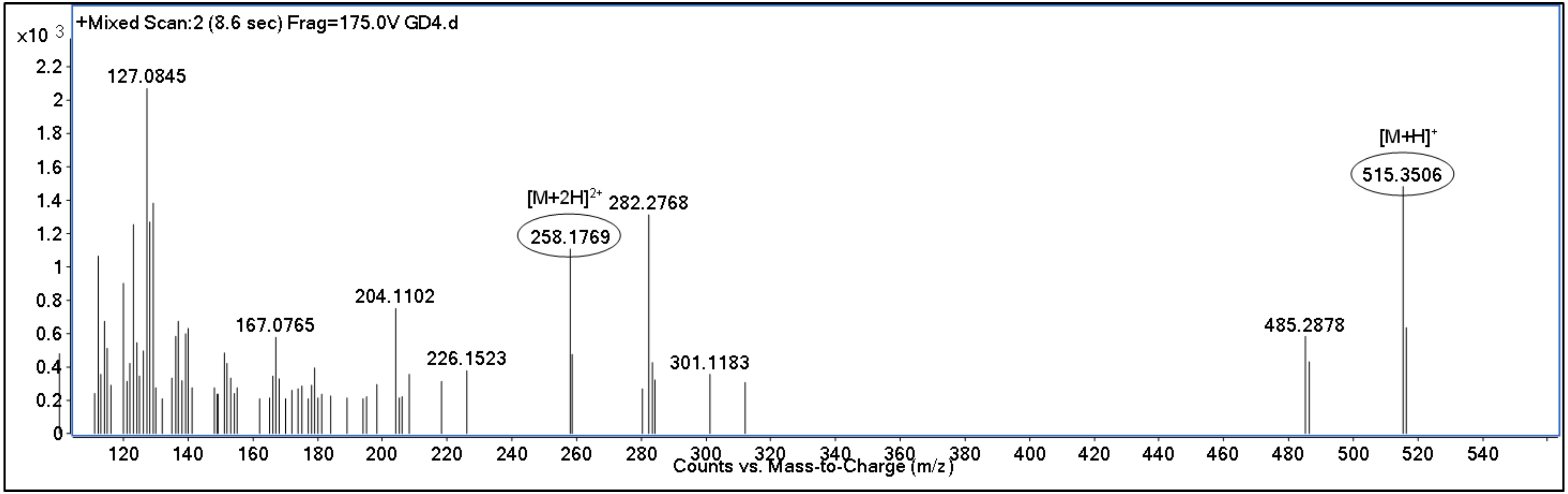

+Mixed Scan:2 (8.6 sec) Frag=175.0V GD4.d
x10 3
127.0845
167.0765
204.1102
226.1523
[M+2H]2+
258.1769
282.2768
301.1183
485.2878
[M+H]+
515.3506
Counts vs. Mass-to-Charge (m/z)

D5

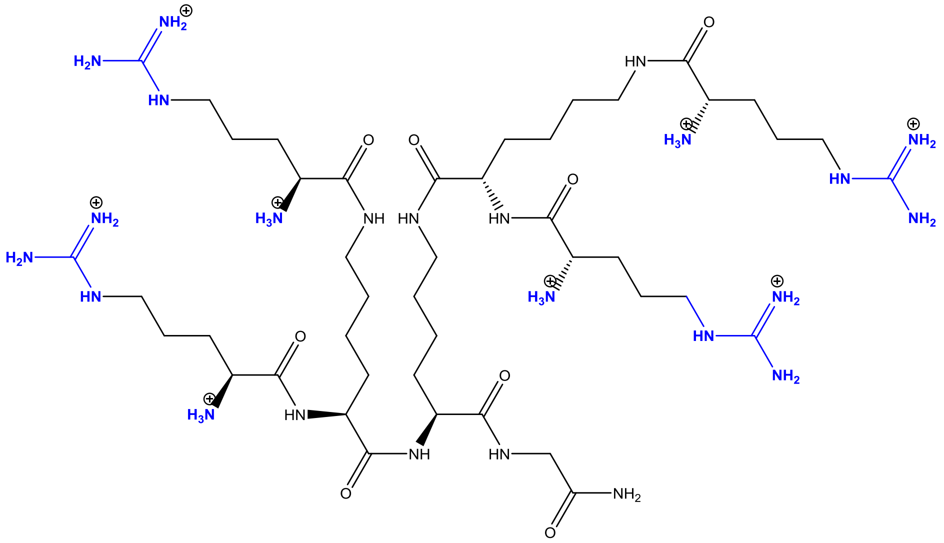

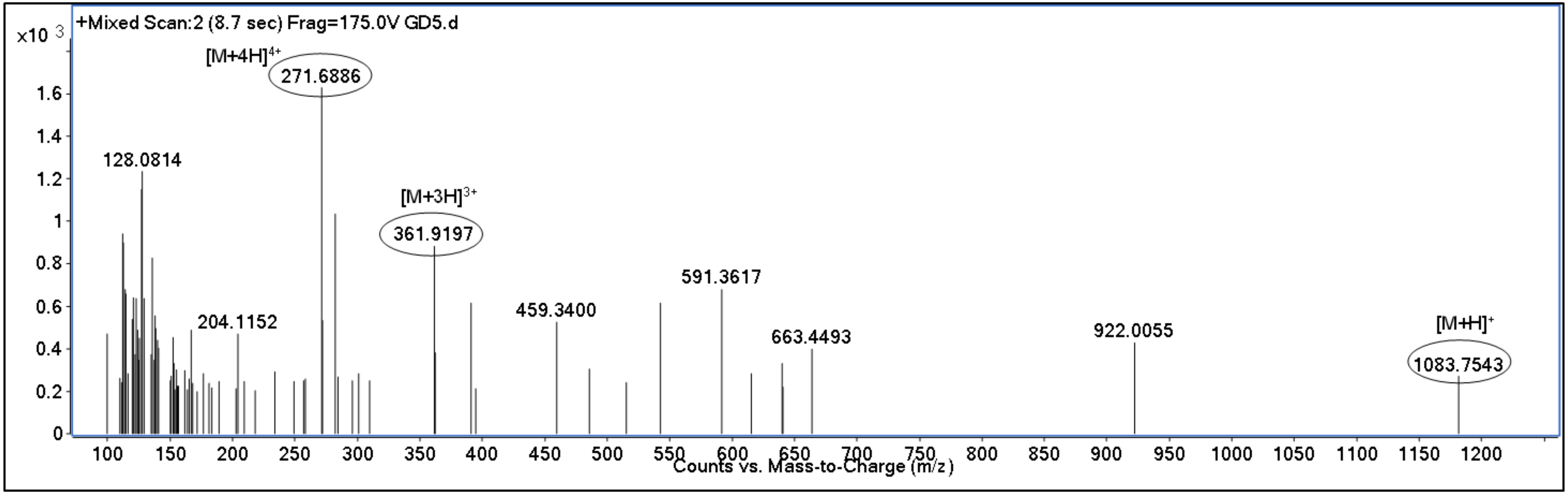

+Mixed Scan:2 (8.7 sec) Frag=175.0V GD5.d
x10 3
[M+4H]4+
271.6886
128.0814
[M+3H]3+
361.9197
204.1152
459.3400
591.3617
663.4493
922.0055
[M+H]+
1083.7543
Counts vs. Mass-to-Charge (m/z)

D6

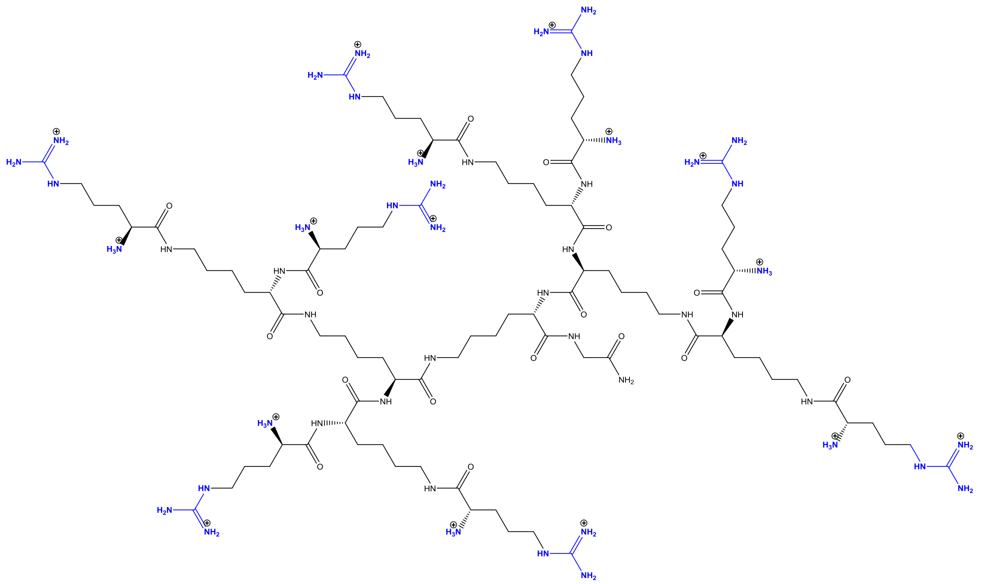

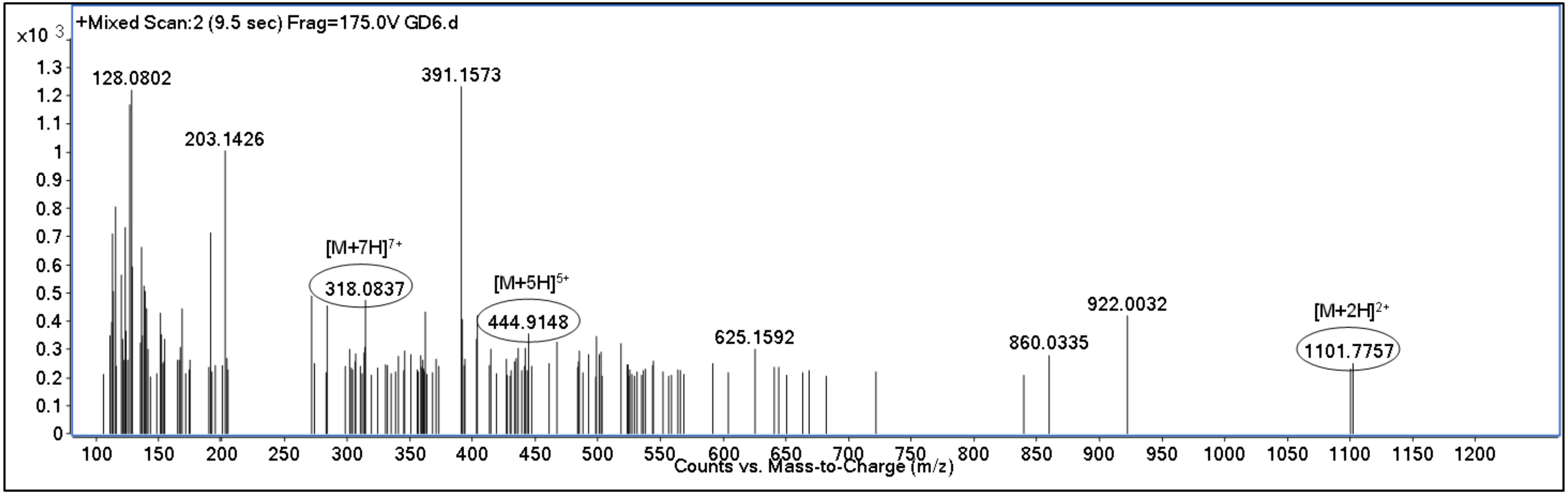
+Mixed Scan:2 (9.5 sec) Frag=175.0V GD6.d
x10 3
128.0802
203.1426
391.1573
[M+7H]7+
318.0837
[M+5H]5+
444.9148
625.1592
860.0335
922.0032
[M+2H]2+
1101.7757
Counts vs. Mass-to-Charge (m/z)

D7

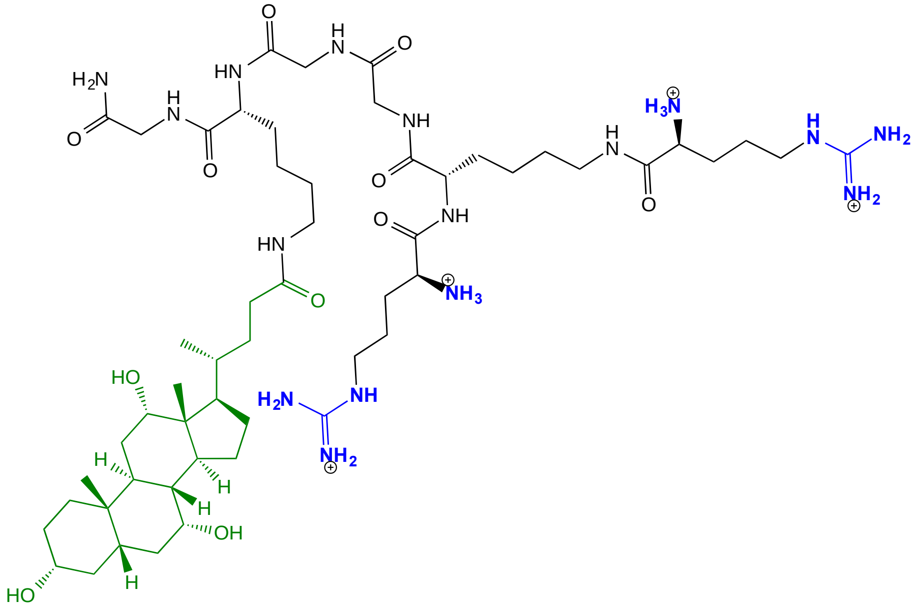
H2N
O
H
N
O
HN
O
H
N
O
NH
O
NH
H3N
H
N
O
H
N
NH2
NH2
HN
O
O
NH3
HO
H2N
NH
NH2
H
H
H
OH
HO
H

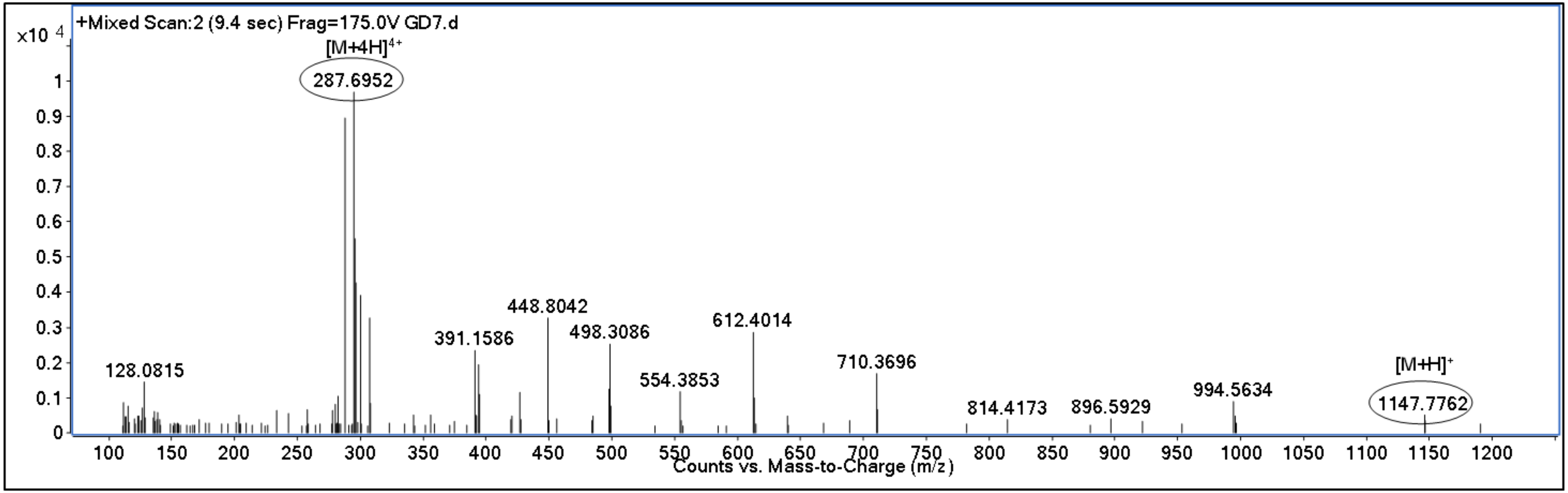
x10 4
+Mixed Scan:2 (9.4 sec) Frag=175.0V GD7.d
[M+4H]4+
287.6952
128.0815
391.1586
448.8042
498.3086
554.3853
612.4014
710.3696
814.4173
896.5929
994.5634
[M+H]+
1147.7762
Counts vs. Mass-to-Charge (m/z)

D8

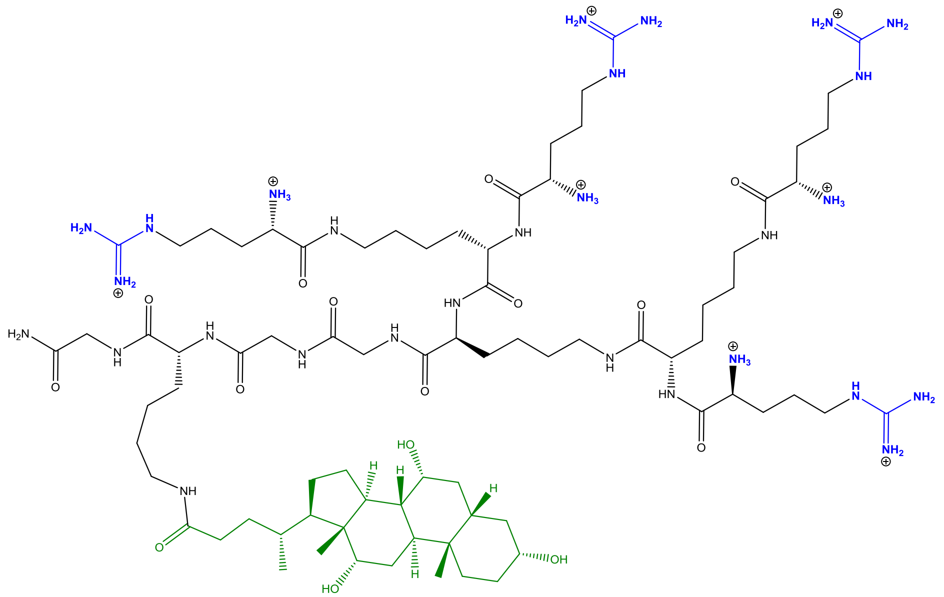

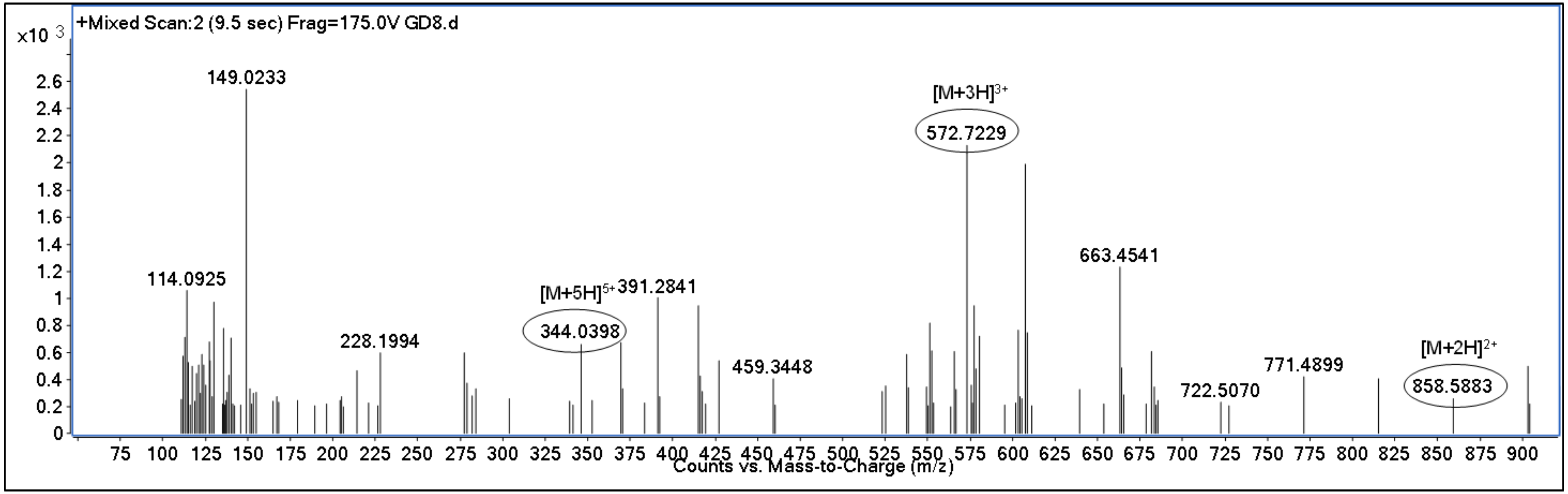

x10 3
+Mixed Scan:2 (9.5 sec) Frag=175.0V GD8.d
149.0233
[M+3H]3+
572.7229
663.4541
114.0925
[M+5H]5+
391.2841
344.0398
228.1994
459.3448
771.4899
722.5070
[M+2H]2+
858.5883
Counts vs. Mass-to-Charge (m/z)

D9

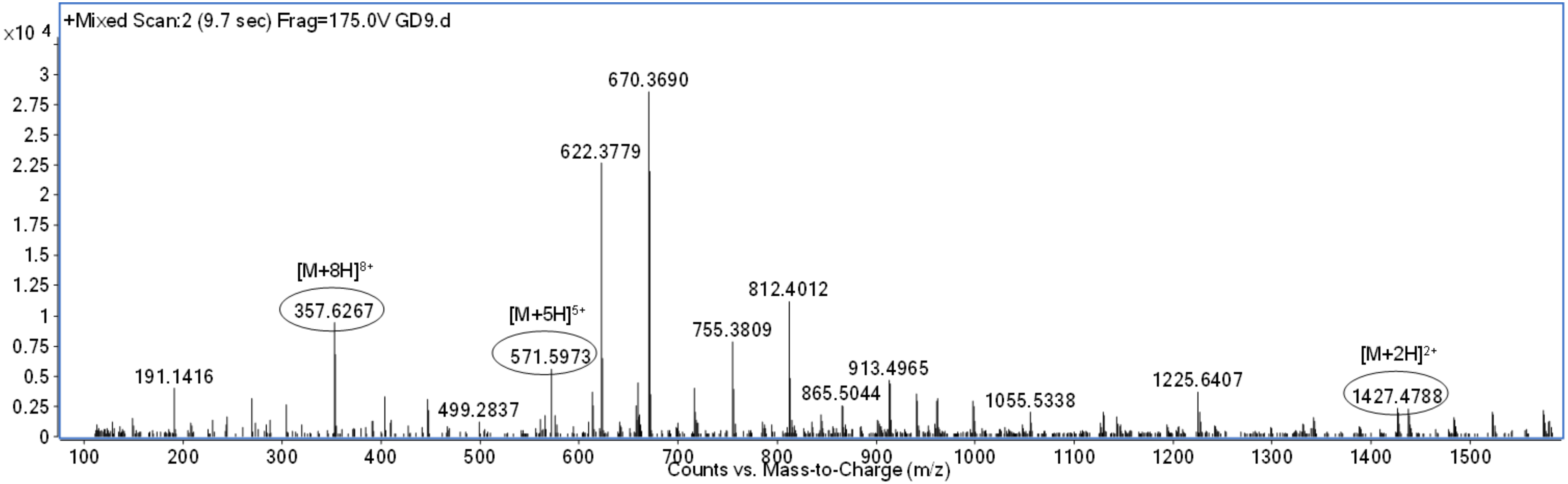

+Mixed Scan:2 (9.7 sec) Frag=175.0V GD9.d
x10 4
670.3690
622.3779
[M+8H]8+
357.6267
[M+5H]5+
571.5973
812.4012
755.3809
191.1416
913.4965
865.5044
1055.5338
1225.6407
[M+2H]2+
1427.4788
499.2837
Counts vs. Mass-to-Charge (m/z)

D10

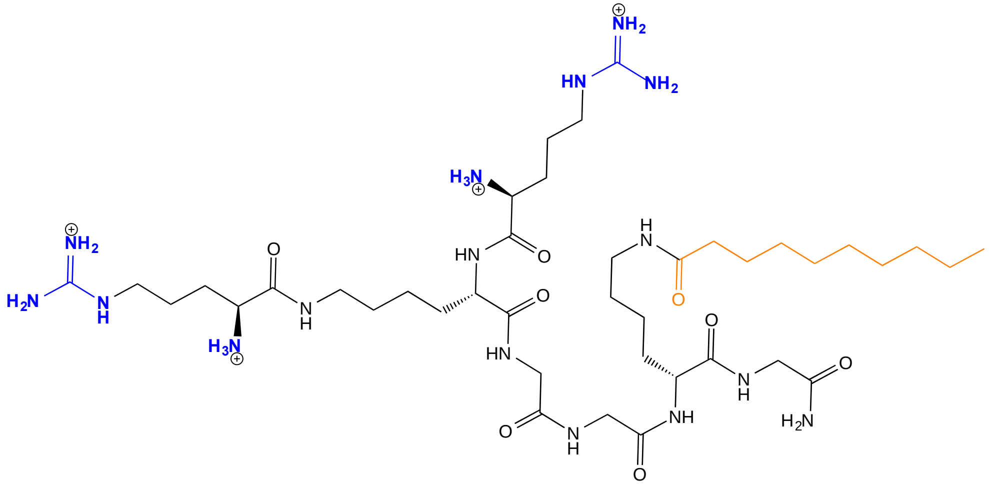

NH2
HN
NH2
H3N
H
N
O
NH2
H2N
N
H
O
HN
N
H
H3N
O
O
HN
O
O
N
H
N
H
O
NH
H2N
O

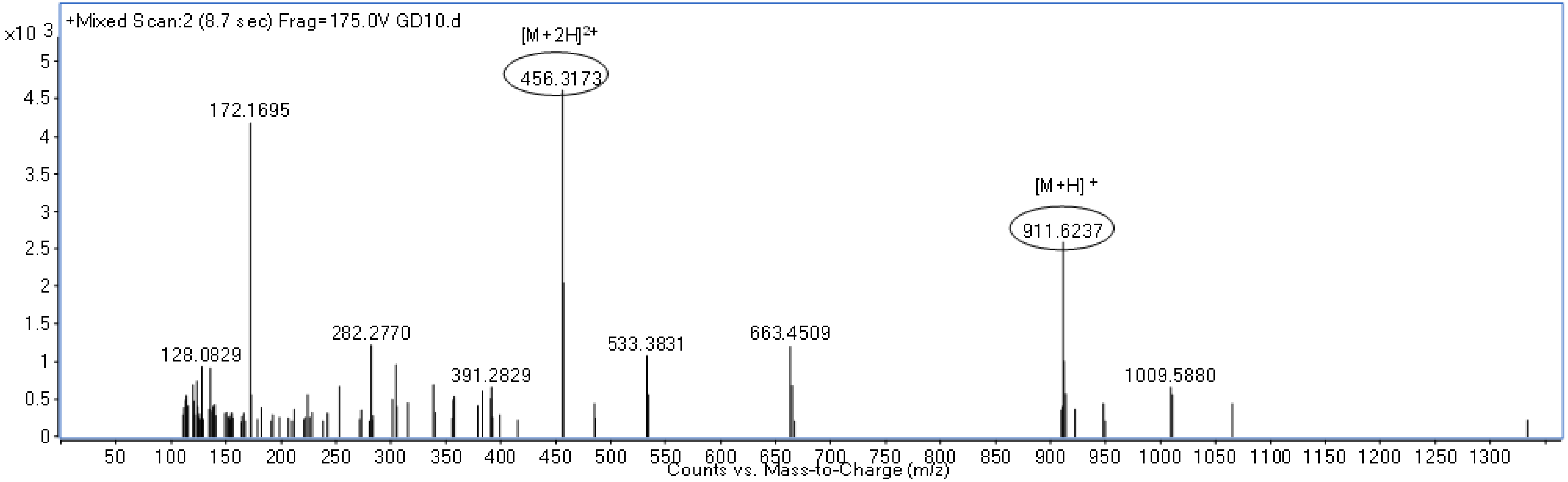

x10 3
+Mixed Scan:2 (8.7 sec) Frag=175.0V GD10.d
[M+2H]2+
456.3173
172.1695
[M+H] +
911.6237
128.0829
282.2770
391.2829
533.3831
663.4509
1009.5880
Counts vs. Mass-to-Charge (m/z)

D11

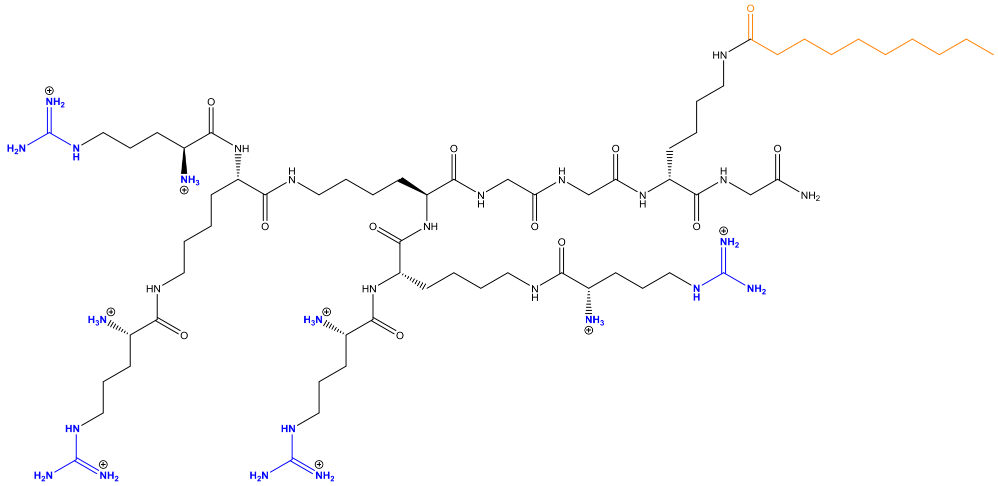

HN
O
NH2
H2N
N
H
NH
NH3
O
H
N
O
N
H
O
H
N
O
N
H
H
N
O
NH2
O
NH
O
HN
NH2
NH2
H3N
HN
O
H3N
HN
O
N
H
NH3
N
H
HN
H2N
NH2
HN
H2N
NH2

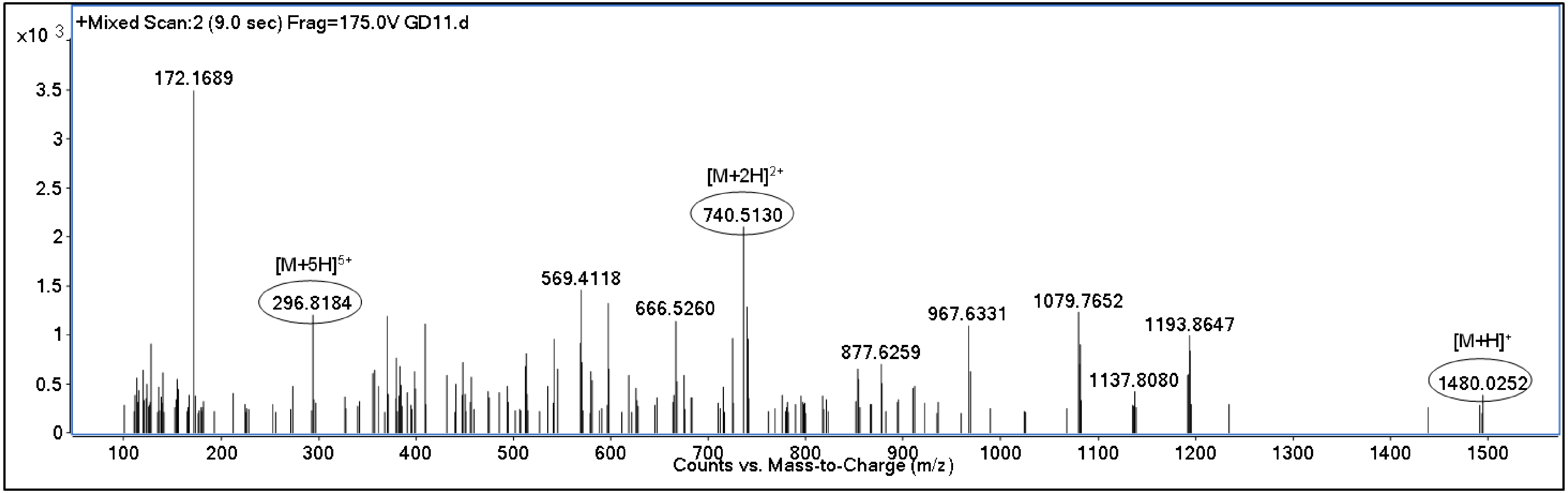

+Mixed Scan:2 (9.0 sec) Frag=175.0V GD11.d
x10 3
172.1689
[M+2H]2+
740.5130
[M+5H]5+
296.8184
569.4118
666.5260
967.6331
1079.7652
1193.8647
877.6259
1137.8080
[M+H]+
1480.0252
Counts vs. Mass-to-Charge (m/z)

D12

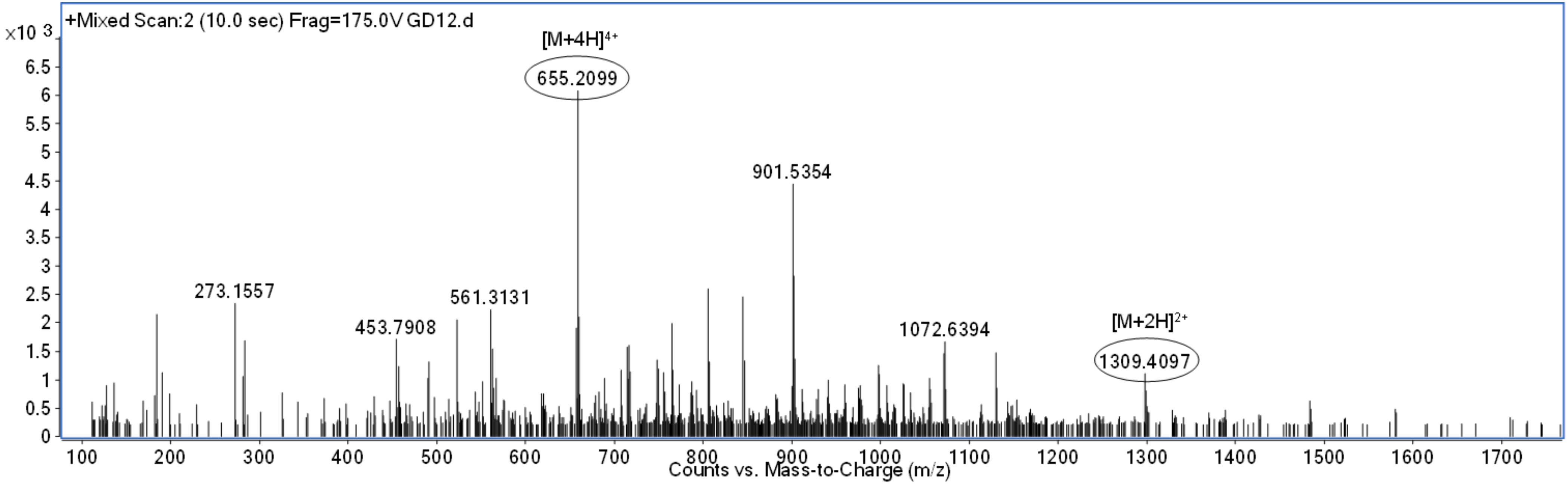
+Mixed Scan:2 (10.0 sec) Frag=175.0V GD12.d
x10 3
[M+4H]4+
655.2099
901.5354
273.1557
561.3131
453.7908
1072.6394
[M+2H]2+
1309.4097
Counts vs. Mass-to-Charge (m/z)
